# Manipulating Fatty Acid Biosynthesis in Microalgae for Biofuel through Protein-Protein Interactions

**DOI:** 10.1371/journal.pone.0042949

**Published:** 2012-09-13

**Authors:** Jillian L. Blatti, Joris Beld, Craig A. Behnke, Michael Mendez, Stephen P. Mayfield, Michael D. Burkart

**Affiliations:** 1 Department of Chemistry and Biochemistry, University of California San Diego, La Jolla, California, United States of America; 2 Sapphire Energy Inc., San Diego, California, United States of America; 3 Division of Biological Sciences, University of California San Diego, La Jolla, California, United States of America; Argonne National Laboratory, United States

## Abstract

Microalgae are a promising feedstock for renewable fuels, and algal metabolic engineering can lead to crop improvement, thus accelerating the development of commercially viable biodiesel production from algae biomass. We demonstrate that protein-protein interactions between the fatty acid acyl carrier protein (ACP) and thioesterase (TE) govern fatty acid hydrolysis within the algal chloroplast. Using green microalga *Chlamydomonas reinhardtii* (Cr) as a model, a structural simulation of docking CrACP to CrTE identifies a protein-protein recognition surface between the two domains. A virtual screen reveals plant TEs with similar *in silico* binding to CrACP. Employing an activity-based crosslinking probe designed to selectively trap transient protein-protein interactions between the TE and ACP, we demonstrate *in vitro* that CrTE must functionally interact with CrACP to release fatty acids, while TEs of vascular plants show no mechanistic crosslinking to CrACP. This is recapitulated *in vivo*, where overproduction of the endogenous CrTE increased levels of short-chain fatty acids and engineering plant TEs into the *C. reinhardtii* chloroplast did not alter the fatty acid profile. These findings highlight the critical role of protein-protein interactions in manipulating fatty acid biosynthesis for algae biofuel engineering as illuminated by activity-based probes.

## Introduction

In our quest to replenish diminishing reserves of fossil fuels with high energy alternatives while mitigating CO_2_ emissions, microalgae have emerged as an attractive option to convert solar energy directly into fungible fuels [1]. However, for microalgal biofuels to be used at industrial scale, productivity must be enhanced and consistency made tunable [2]. For example, to develop biodiesels to function in existing petroleum-based infrastructure, fatty acids from microalgae must be altered to more closely mimic conventional diesel [3]. Fusion of the powerful tools of systems biology and metabolic engineering could enable us to develop microalgal strains capable of producing commercially viable quantities fatty acids with desired chain lengths [4]. New advances in algal genetic engineering will allow us to fashion viable fuels and commodities from microalgal metabolic pathways [5], [6], yet our knowledge of algal fatty acid biosynthesis remains incomplete. Without a detailed understanding of enzyme activity, timing, and regulation, the engineering of biofuel products from these pathways will struggle to meet our growing energy demands.

Fatty acid biosynthesis has been successfully manipulated in oilseed crops to produce fatty acids with novel compositions [7]. In pioneering work, Voelker and coworkers achieved short circuiting of fatty acyl chain elongation by expressing a laurate (12:0)-specific thioesterase from the California bay plant (*Umbellularia californica*) in the seeds of *Arabidopsis* and rapeseed *(Brassica napus)* to increase laurate by 24 and 58%, respectively [8]. Since the discovery that heterologous expression of thioesterases can influence the lipid profile of an organism [8], plant TEs have been engineered into a variety of plant species effectively altering their oil content [7].

By terminating fatty acid biosynthesis, the TE functionally determines the length and identity of the fatty acid end product [9]. Plant FatA TEs select for oleoyl (18:1)-ACP substrates and FatB TEs preferentially hydrolyze ACPs loaded with saturated fatty acids [10]. Some plants have evolved FatB TEs capable of prematurely siphoning short chain fatty acids for incorporation into seed storage oil [11]. Of the range of fatty acids found in Nature, saturated medium chain fatty acids (C_8_–C_14_) are ideal for biodiesel because they have properties that mimic current diesel fuels [4]. Recently, plant FatB TEs were genetically engineered into diatoms (*Phaeodactylum tricornutum*) [12] and cyanobacteria (*Synechocystis* sp. PCC6803) [13] with the goal of creating a superior biodiesel feedstock, but these efforts were met with limited success.

*De novo* fatty acid biosynthesis occurs within an algal plastid by action of a type II fatty acid synthase (FAS), a modular multi-domain enzymatic complex where each activity is encoded onto a separate protein [14]. Central to FAS, an acyl carrier protein (ACP) acts as a metabolic scaffold, tethering the growing fatty acid as it is shuttled iteratively between catalytic domains of the synthase. Fatty acid biosynthesis begins by post-translational modification of the ACP catalyzed by a phosphopantetheinyl transferase (PPTase), which transfers 4′-phosphopantetheine from coenzyme A to a conserved serine residue on ACP. This converts inactive *apo*-ACP to its active *holo* form bearing a flexible prosthetic arm for attachment of fatty acids via thioester linkage. Once *holo*-ACP is loaded with an acyl starter unit, fatty acid synthesis occurs on the ACP by sequential action of ketosynthase (KS), ketoreductase (KR), dehydratase (DH), and enoyl reductase (ER) enzymes, each cycle resulting in a net addition of two carbons to the growing chain. During chain elongation, ACP buries the growing fatty acid in its hydrophobic core to protect it from hydrolysis [15]. Functional interaction with each FAS domain induces a conformational change in ACP that draws the acyl chain out of the pocket (‘switchblade’ mechanism) for further processing by downstream enzymes [16]. When a mature fatty acid has been assembled on the ACP, a thioesterase (TE) catalyzes acyl transfer from ACP to its active site cysteine residue, priming the thioester for attack by water. Based on our previous work in bacteria [17] as well as plant studies to manipulate fatty acid chain length [7], we propose that in algal fatty acid biosynthesis, the TE must functionally interact with ACP to receive the acyl chain, and thus significant protein-protein interactions between the ACP and TE are required to mediate the hydrolysis of fatty acids.

We chose to investigate *Chlamydomonas reinhardtii*, as it is one of a small number of algal species for which all three genomes have been sequenced [18]. Most importantly, it is one of only ten algae that can be transformed with current genetic technologies [19], and techniques have been developed that facilitate robust expression of transgenes in both the nuclear and plastid genome of *C. reinhardtii* [20], [21]. Together, these make *C. reinhardtii* an ideal model organism to explore algal fatty acid biosynthesis and decipher ACP-TE interactions. Such dynamic protein-protein interactions within the cell are well-known to govern many biological processes [22], [23]. Typical methods to decipher protein-protein interactions include yeast 2-hybrid and TAP-tag systems [24], although these techniques can produce false positive readings. Chemical crosslinking can also be used to identify partner proteins, but this procedure suffers from a lack of specificity [24]. To investigate ACP-TE interactions *in vitro*, we employ a novel approach that circumvents issues associated with traditional methods. Activity-based chemical crosslinking is a powerful means to capture the transient interactions occurring between FAS domains and ACP [17]. We have used this methodology extensively in prokaryotic systems to study protein-protein interactions, successfully implementing activity-based probes designed for a wide array of activities [25], [26].

Here, we illustrate the use of activity-based probes to investigate ACP-TE interactions towards engineering fatty acid chain length in *C. reinhardtii*. By employing chemical probes specifically designed for the enzymatic activity of TEs, we report the first demonstration of metabolic product control through protein-protein interactions. This work emphasizes the importance of protein-protein recognition between the ACP and catalytic enzymes in mediating fatty acid biosynthesis in algal plastids. We address this hypothesis *in silico*, *in vitro* and *in vivo* using *C. reinhardtii* as a model system. Characterizing algal FAS domains and their interactions will further enable us to optimize heterologous expression of fatty acid biosynthetic enzymes in microalgae and significantly alter the fatty acid profile of transgenic production strains.

## Results

### Sequence alignment of plant TEs and comparison to CrTE

**Figure 1:**
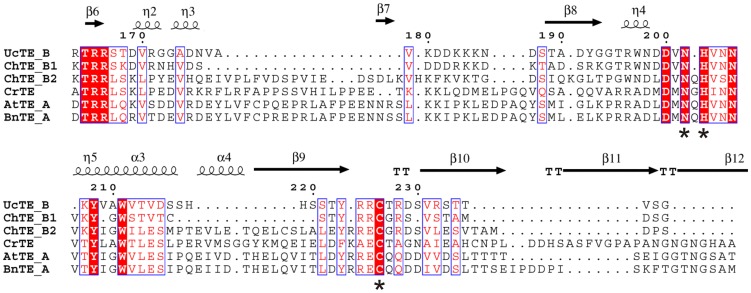
Thioesterase sequence alignments. Structure-based sequence alignment of FatA TEs from *Arabidopsis thaliana* (AtTE_A) and *Brassica napus* (BnTE_A), *Chlamydomonas reinhardtii* (CrTE), and FatB TEs from *Cuphea hookeriana* (ChTE_B1 and ChTE_B2) and *Umbellularia californica* (UcTE_B). The Cys-His-Asn catalytic triad is shown by an asterisk (*). Conserved residues are highlighted in red and similar residues in blue boxes.

To critically compare the *C. reinhardtii* TE sequence to activity and its structure to function, we first conducted a multiple sequence alignment between plant FatA and FatB TEs and CrTE (Figs. 1, S1). Structure-based analysis of known TEs and comparison to CrTE places it in thioesterase family TE14 along with FatA and FatB TEs [27]. Sequence alignment with plant TEs shows that CrTE contains a catalytic cysteine residue as part of a Cys-Asn-His catalytic triad (Fig. 1). FatB TEs have a conserved hydrophobic 18-amino acid domain [28], while FatA TEs contain distinctive sub-motifs in an N-terminal 60 amino acid transit peptide sequence [29]. Although ChloroP1.1 identifies a 60 amino acid plastid targeting sequence at its N-terminus (Fig. S1), CrTE is distinct from FatA and FatB TEs in this region, which has been proposed as the source for substrate specificity in TEs [9]. BLAST analysis indicates that CrTE shares the closest homology to predicted TEs from *Volvox carteri* and *Chlorella variabilis*, a multicellular chlorophyte alga and a unicellular green alga, respectively, both closely related to *C. reinhardtii*.

An exhaustive search of medium chain-specific plant TEs for homology to the CrTE resulted in a panel of fourteen plant TEs (Text S1). Because there are currently no crystal structures available for plant or algal TEs, homology models were constructed, and TEs were screened computationally for functional binding to CrACP.

### *In silico* modeling of ACP-TE protein-protein interactions and TE-substrate interactions

**Figure 2:**
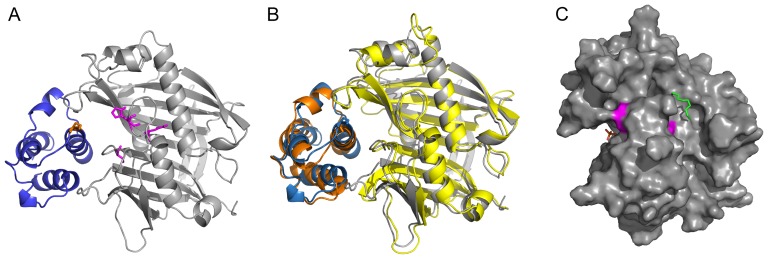
Thioesterase modeling, docking of ACP-TE protein-protein interactions, and blind substrate docking of fatty acid substrate to *C. reinhardtii* TE. (*A*) Docking of CrTE (grey) with Cr-cACP (blue) showing a <10 Å distance between Cr-cACP Ser_43_ (orange) and the active site Cys_306_His_270_Asn_268_ triad (magenta) of CrTE. (*B*) Docked complex of CrTE (grey) and ChTE (yellow) showing similar binding modes of Cr-cACP to both plant and algal thioesterases. (*C*) Surface representation of blind docking of stearyl-4′-phosphopantetheine to CrTE showing the thioester bond of the substrate in close proximity to the TE active site and stearate in the binding tunnel of CrTE.

While there are no plant or algal TE structures deposited in the PDB, two crystal structures (PDB: 2OWN and 2ESS) display homology and high sequence identity (37.9% and 41.4%, respectively) to CrTE around the active site residues Cys-His-Asn (8 Å). The modeled CrTE obtained from two different homology algorithms are nearly superimposable and in line with previous modeled TE structures including those from *Arabidopsis* [30] and *Jatropha* [29]. To investigate the binding mode of a fatty acid by CrTE and its substrate specificity, the modeled CrTE was subjected to non-biased docking of a stearate-pantetheine substrate (Fig. S2). Consistently, the calculated top 10 models display the model with pantetheine substrates bound to CrTE in its binding tunnel (Fig. 2C) with the thioester exposed to the tentative active site. Experimentally observed fatty acyl substrates (14:0, 16:0, 16:4, 18:0, 18:1 and 18:3) were docked onto the model structure of CrTE and all fatty acids dock to the binding tunnel of CrTE (Fig. S2). However, the 16:4 and 18:3 substrates do not bind in a favorable orientation, as expected since desaturations are typically derived from post-synthase desaturase enzymes.

*C. reinhardtii* chloroplastic ACP (Cr-cACP) was modeled and docked onto TEs using the Cluspro server (Fig. 2). We generated the homology model of Cr-cACP based on the template of the ACP from *Aquifex aeolicus* (PDB: 2EHTA), which shows a sequence identity of 52.6%, a q-meanscore of 0.7, and a residual RMSD of 0.08 between template and model. Models were compared with those obtained from the i-Tasser server [31] and no significant deviations between the two homology modeling algorithms were observed. The models generated from protein-protein docking were examined for binding of *apo*-ACP close to the TE active site and for juxtaposition of the ACP serine in relation to the TE active site cysteine. Docking of Cr-cACP to CrTE displayed large surface interactions between helix II of Cr-cACP and the CrTE as well as a productive orientation of the ACP serine to TE cysteine (Fig. 2A), consistent with previous studies of protein-protein interactions between ACP and partner FAS enzymes [32], [33]. Examining electrostatic potential maps of Cr-cACP/CrTE models shows electrostatic complementarity, and docking predicts a 13.5 Å distance between Cr-cACP conserved serine and CrTE active site cysteine residue (Fig. S5).

Virtually screening a panel of fourteen vascular plant TEs for interaction with Cr-cACP using a high-end computational cluster collecting data over several months [34] for each TE, *U. californica* TE (UcTE) and *C. hookeriana* TE (ChTE) emerged as promising candidates based on their ability to dock in proximity to the Cr-cACP Ser43 (Figs. 2B, S3). Careful examination of Cr-cACP with UcTE and ChTE revealed that these ACP-TE models closely resemble the Cr-cACP/CrTE model, indicating a possible productive interaction between Cr-cACP and plant TEs. In contrast, modeling *C. reinhardtii* mitochondrial ACP (Cr-mACP) with these same plant TEs shows poor docking, suggesting that not all algal ACPs will successfully bind, and thus not catalytically interact, with plant TEs (Figs. S4, S5). Although the electrostatic potential map of Cr-mACP is similar to Cr-cACP, docking Cr-mACP to CrTE illustrates a different binding motif than that of Cr-cACP/CrTE, and as a result, the distance between Cr-mACP serine and CrTE cysteine is predicted to be 18.8 Å (Fig. S5).

### Characterization of fatty acid acyl carrier proteins of *C. reinhardtii*

**Figure 3:**
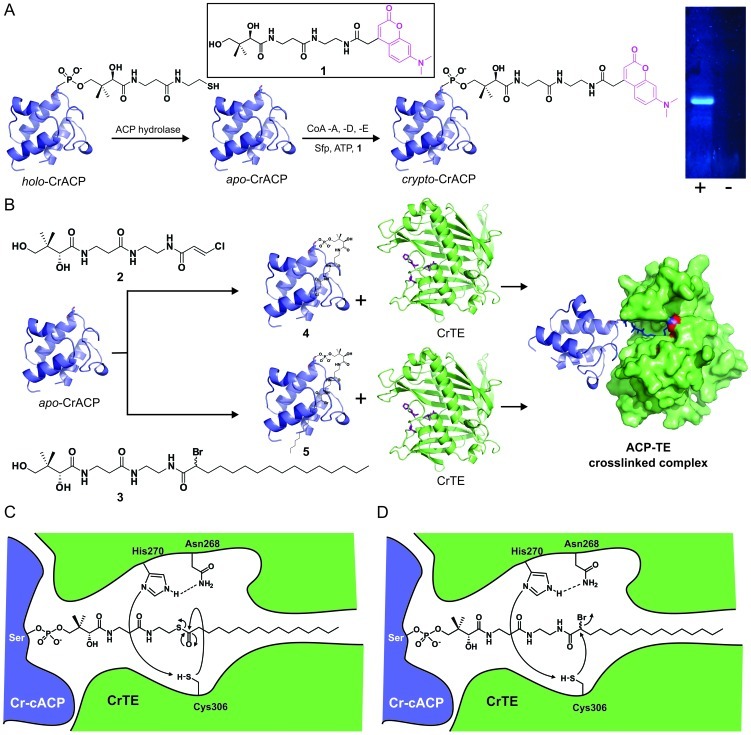
Schematic of activity-based crosslinking between CrACP and TEs. (*A*) *Apo*-CrACP is formed by treating *holo*-CrACP with ACP hydrolase from *P. aeruginosa* [36], removing the pantetheine moiety from the conserved serine of CrACP. Presence of *apo*-CrACP is confirmed using a one-pot fluorescent labeling method [37], detected by visualization of a resulting SDS-PAGE gel at 365 nm. (+) formation of fluorescent *crypto*-CrACP; (−) control reaction in which fluorescent pantetheine analogue **1** was omitted. (*B*) Activity-based crosslinking scheme. *Apo*-CrACP is incubated with **2** or **3**, Sfp, ATP, CoA-A, CoA-D, and CoA-E to generate the corresponding *crypto*-CrACPs **4** and **5**. Upon incubation of *crypto*-CrACP with TE, protein-protein interactions trigger a site-specific covalent crosslinking reaction with the chloroacrylamide in **4** or the α-bromoamide in **5**, forming an ACP-TE crosslinked complex. (*C*) Predicted enzymatic mechanism of the hydrolytic release of a fatty acid by CrTE using a Cys-Asn-His catalytic triad. (*D*) Mechanism of irreversible crosslink between TE and *crypto*-CrACP containing a reactive bromide on the carbon α to the site of nucleophilic attack by the TE.

To study ACP-TE interactions *in vitro*, we obtained both fatty acid ACPs identified in the *C. reinhardtii* genome, ACP1 and ACP2. Based upon alignment with plant mitochondrial and chloroplastic ACPs, homology, and BLAST analysis, ACP1 and ACP2 correspond to the mitochondrial and chloroplastic ACPs, respectively (Fig. S6, Text S1). ACPs were synthesized in *E. coli* codon bias and cloned into pET28b expression vectors bearing an N-terminal-His_6_ tag (Table S1). Whereas Cr-mACP (ACP1) formed insoluble aggregates when expressed in *E. coli* and required refolding [35], Cr-cACP (ACP2) was expressed in *E. coli* in soluble form. Because we anticipated a stronger interaction between the ACP of the chloroplast and the CrTE, Cr-cACP was used as a positive control in crosslinking experiments (for details about Cr-mACP, we refer to Text S1). After Ni-NTA purification, a mixture of *apo*-Cr-cACP and *holo*-Cr-cACP (Fig. 3A) was obtained. The conversion of *holo*-Cr-cACP to *apo*-Cr-cACP was catalyzed by ACP-hydrolase (ACPH) from *Pseudomonas aeruginosa* [36], which removed the 4′-phosphopantetheine moiety from Ser43 of Cr-cACP (Figs. 3A, S7). The formation of *apo*-Cr-cACP was confirmed by mass spectrometry (Fig. S8). A one-pot chemoenzymatic method was used to validate *in vitro* post-translational modification of *apo*-Cr-cACP [37]. As shown in Fig. 3A, incubation of *apo*-Cr-cACP with Sfp, a surfactin synthetase-activating PPTase from *B. subtilis* [38], coenzyme A biosynthetic enzymes (CoA-A, CoA-D, and CoA-E), ATP, and fluorescent pantetheine analogue **1** resulted in the formation of fluorescently-labeled *crypto*-Cr-cACP. This conversion validated proper folding and activity of Cr-cACP and confirmed our ability to successfully load the activity-based probe onto ACP for further studies of protein-protein interactions [39].

### Expression, purification, and activity of Cr, Uc, and Ch thioesterases

CrTE, UcTE, and ChTE genes were synthesized in *E. coli* codon bias and subcloned into pET21a expression vectors bearing a C-terminal FLAG tag (Table S1). Expression in *E. coli* and FLAG affinity chromatography yielded pure TE protein. After reduction, plant and algal thioesterase activity was confirmed kinetically by monitoring hydrolysis of para-nitrophenylhexanoate [40].

### *In vitro* crosslinking assay to screen TEs for functional binding to Cr-cACP and design of thioesterase substrate mimic

Recently, we developed a crosslinking assay based on enzymatic activity that interrogates specific protein complex formation in FAS, PKS, and NRPS modular synthases using pantetheine probes [26]. Work in our laboratory has demonstrated that activity-based crosslinking serves as a quantitative measure of the strength of functional binding interactions between ACP and modular FAS domains [41]. In this assay, selectivity arises from protein-protein interactions, rather than purely protein-substrate specificity, as shown by examining biochemically unrelated carrier proteins modified with the same alkylating functionality [26]. This method is far more selective than traditional techniques aimed at interpreting protein-protein interactions, thus preventing false positive readings from non-specific protein complex formation.

To investigate ACP-TE interactions, we sought to design a chemical probe that would selectively react with the active site of the TE. Chloroacrylic pantetheine probe **2** was used in previous studies to generate a site-specific crosslink between *E. coli* ACP and *E. coli* KS, triggered by protein-protein recognition [26]. Based on sequence alignment (Fig. 1), the CrTE uses an active site cysteine as its catalytic residue during fatty acid biosynthesis, similar to the KS domain. However, the TE is fundamentally different from the KS, as it only acts upon the final acyl-ACP substrate, whereas the KS acts on intermediate chain lengths. When a mature fatty acid is assembled on the ACP, the TE first catalyzes acyl transfer to its active site cysteine residue (Fig. 3C). The product is then hydrolyzed as water attacks the pendant thioester to yield a free fatty acid. Modeling studies of CrTE illustrate a shallow hydrophobic tunnel at the surface of the enzyme, where the acyl chain presumably lies (Fig. 2C). Previously, a panel of pantetheine probes was used to examine the compatibility of ACP-KS partners, which established that substrate recognition also plays a critical role in catalysis [25]. Hypothesizing that the fatty acyl chain factors into enzymatic hydrolysis by the TE, we designed an activity-based substrate mimic containing a reactive bromide α to the site of nucleophilic attack by the TE and a long aliphatic tail (Fig. 3D). To test the substrate specificity and selectivity of the CrTE using activity-based probes, we designed a shorter α-bromohexyl pantetheine analogue composed of only six carbons (**6**). Chemical synthesis of α-bromopalmitic pantetheine probe **3** and its shorter analogue **6** (Text S1) enabled us to study ACP-TE interactions using three different activity-based probes (**2, 3,** and **6**).

**Figure 4:**
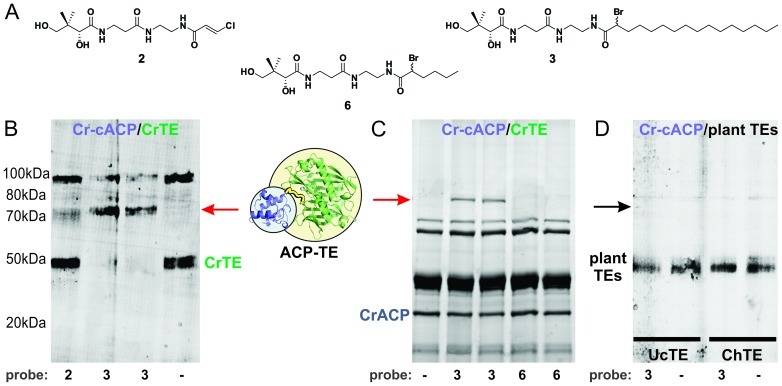
Activity-based crosslinking as a determinant of functional interaction with *C. reinhardtii* cACP. (*A*) Activity-based substrate mimics used in crosslinking assay; (*B*) SDS-PAGE gel showing Cr-cACP/CrTE interaction. *Apo*-Cr-cACP was modified with pantetheine analogue **2** or **3** to generate the corresponding *crypto*-Cr-cACPs (**4** and **5**). *Crypto*-Cr-cACPs were incubated with CrTE and crosslinking was visualized by SDS-PAGE analysis following FLAG affinity purification of the CrACP/TE complex. The band observed at 50 kDa is the FLAG-tagged CrTE and the band detected at ∼75 kDa is the ACP/TE complex. During overnight incubation at 37°C, reduced CrTE shows spontaneous oxidation (bands at ∼100 kDa). Pantetheine analogues used in crosslinking reactions are noted under the gel. A red arrow illustrates the ACP-TE complex in (*B*) and (*C*). (*C*) *Apo*-Cr-cACP was reacted with either α-bromopalmitic pantetheine probe **3** or α-bromohexyl pantetheine probe **6** to generate *crypto*-Cr-cACPs with C16 and C6 acyl chains attached, respectively. Each *crypto*- Cr-cACP was incubated with CrTE and monitored for extent of crosslinking by SDS-PAGE analysis of Ni-NTA-purified reactions. (*D*) *Apo*-Cr-cACP was modified with α-bromopalmitic pantetheine analogue **3** to form *crypto*-Cr-cACP, which was incubated with UcTE (left 2 lanes) or ChTE (right 2 lanes). Crosslinking was measured by SDS-PAGE analysis following FLAG affinity purification. The bands at ∼50 kDa are plant TEs.

Manipulating the CoA biosynthetic pathway [39], functionalized CoA analogues were formed *in vitro* from chloroacrylamide **2** or α-bromopalmitamide **3** (Fig. 3B). Sfp [38] was used to attach substrate mimics to Ser43 of *apo*-Cr-cACP, generating reactive *crypto*-Cr-cACPs **4** and **5** (Fig. 3B) [37]. A crosslinked complex was observed between *crypto*-Cr-cACP and CrTE using both probes, indicating a positive interaction between the Cr-cACP and CrTE (Fig. 4B). Importantly, incubation of α-bromopalmitic *crypto*-Cr-cACP **5** with CrTE resulted in full conversion of CrTE to a Cr-cACP/CrTE complex, indicating a strong functional interaction between the two proteins (Fig. 4B). In contrast, reacting chloroacrylic *crypto*-Cr-cACP **4** with CrTE did not result in a high yield of ACP/TE protein complex formation (Fig. 4B).

To study the effect of the length of the acyl chain attached to Cr-cACP on functional binding to CrTE, a shorter α-bromohexyl pantetheine probe **6** (Text S1) was used for activity-based crosslinking. In agreement with its predicted natural activity, a higher quantitative yield of crosslinked complex was detected between CrTE and longer chain α-bromopalmitic-ACP (Fig. 4C), indicating that chain length factors into enzymatic hydrolysis by the TE.

Based on the results of our initial computational docking screen of plant TEs, UcTE and ChTE were tested for their ability to crosslink with *crypto*-Cr-cACP. Contrary to modeling studies, no crosslink was detected between heterologous plant TEs and Cr-cACP due to an absence of protein-protein recognition (Fig. 4D). *Crypto*-Cr-cACPs formed from either chloroacrylic pantetheine probe **2**, α-bromopalmitic pantetheine probe **3**, or α-bromohexyl pantetheine probe **6** did not result in a crosslinked complex, indicating vascular plant TEs did not functionally interact with Cr-cACP *in vitro* (Fig. S9).

Interestingly, *C. reinhardtii* mitochondrial ACP (Cr-mACP) did not orient favorably when docked onto CrTE, UcTE or ChTE (Figs. S4, S5). Indeed, a crosslinked complex was not detected between Cr-mACP and the CrTE of the chloroplast, demonstrating selective recognition between ACP and TE domains (Fig. S10).

### Engineering thioesterases into Cr chloroplast and fatty acid analysis of transgenic strains

**Figure 5:**
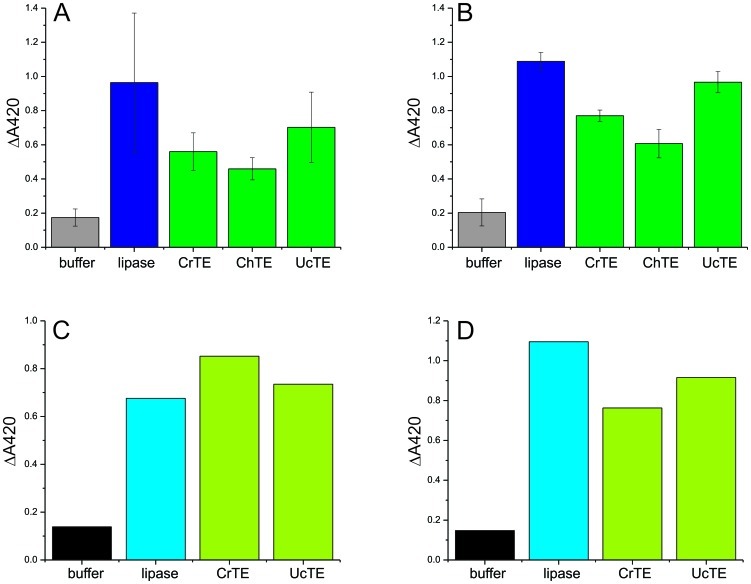
Thioesterase activity assay. Activity of plant and algal thioesterases and porcine pancreas type II lipase were determined by monitoring the hydrolysis of para-nitrophenylhexanoate for 16 hours at 30°C. (*A*) pH 7, TEs expressed in *E. coli*; (*B*) pH 8, TEs expressed in *E. coli*; (*C*) pH 7, TEs expressed in *C. reinhardtii*; (*D*) pH 8, TEs expressed in *C. reinhardtii*.

To determine whether plant TEs functionally interact with the Cr-cACP *in vivo* and to validate our *in vitro* activity-based crosslinking results, CrTE, UcTE, and ChTE were engineered into the *C. reinhardtii* chloroplast (Tables S2, S3). A high level of protein expression was achieved by codon-optimizing the plant TE sequences for the *C. reinhardtii* chloroplast and using endogenous promoters and RNA elements to drive expression [21]. Microprojectile bombardment transformed *C. reinhardtii* strain 137c (mt+) with thioesterase-harboring plastid expression vectors [21]. PCR confirmed integration of the TE genes and homoplasmicity (Fig. S11) (Table S3) and Western blot analysis validated expression of the TEs in the *C. reinhardtii* chloroplast (Fig. S12) [42]. After purification from Cr lysate and reduction, plant and algal thioesterase activity was validated kinetically by monitoring hydrolysis of para-nitrophenylhexanoate (Fig. 5) [40].

**Figure 6:**
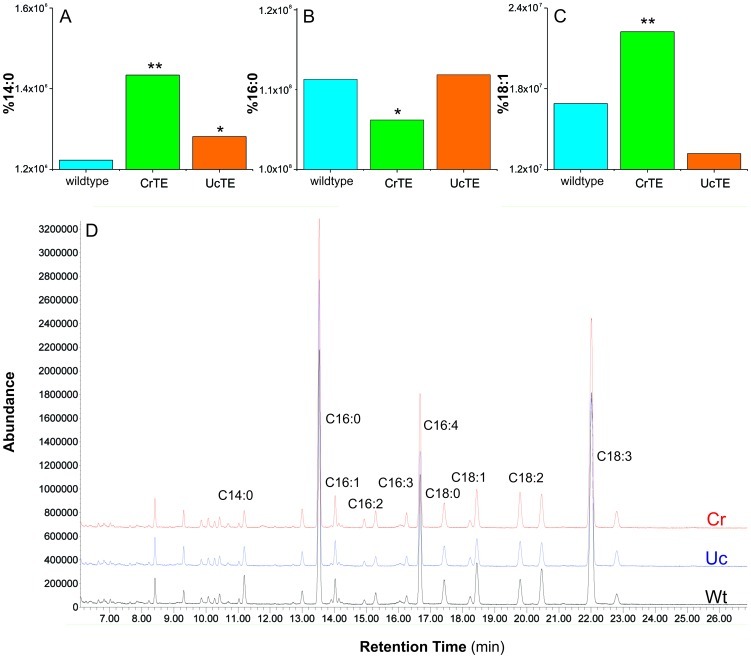
Fatty acid analysis of *C. reinhardtii* strains expressing thioesterases. Fatty acid composition of Cr strains was determined by GC/MS analysis and comparison to authentic standards. Peak areas were integrated and compared to an external standard for quantification. Bar graphs denote abundances of (*A*) Myristic acid (14:0), (*B*) Palmitic acid (16:0), and (*C*) Oleic acid (18:1), and labels on the Y-axis correspond to the percentages of these fatty acids of the total fatty acid content. (*D*) Full GC/MS chromatograms of Cr strains expressing CrTE (red), UcTE (Blue) and wildtype CrTE (black). Three separate cultures of each strain were analyzed for fatty acid content and composition, and data were recorded and averaged with a mean deviation of 7% in each experiment. Statistical analyses were performed using SPSS (v13.0), and for all data analysis, a *p*-value<0.5 was considered statistically significant.

Transgenic algal strains were grown in a greenhouse to late log phase, harvested, and fatty acids converted into their corresponding methyl esters for GC/MS analysis (Text S1). Compared to wildtype, no significant change in fatty acid content or composition was detected in transgenic *C. reinhardtii* strains expressing the UcTE or ChTE (Fig. 6). This was an indication that plant TEs did not functionally interact with Cr-cACP *in vivo* and thus were unable to hydrolyze fatty acids attached to Cr-cACP. Interestingly, the strain overexpressing native CrTE resulted in a short circuiting of fatty acid chain elongation to increase myristic acid (14:0) content by 2.5 fold (*p*-value<0.05) (Fig. 6). These results signify the first time protein-protein interactions have been correlated with product control in algae.

## Discussion

Fatty acid biosynthesis is orchestrated by a modular enzymatic complex within the algal plastid, similar to machinery present in prokaryotes [43]. The type II FAS iteratively condenses 2-carbon acetate units, each cycle resulting in a fully reduced extended carbon chain. All reactions occur with substrates bound as thioesters to a 4′-phosphopantetheine arm attached to ACP. In bacteria, interactions between the ACP and KS are required for proper metabolite assembly [26]. Further studies have established that substrate recognition is also important in KS-mediated condensation [25]. Here, we have shown that significant ACP-TE interactions as well as enzyme-substrate interactions play a role in the hydrolysis of fatty acids within an algal chloroplast.

Docking of Cr-cACP to CrTE identifies a protein-protein recognition surface as well as proximity between the fatty-acyl thioester attached to the Cr-cACP and active site cysteine of CrTE (Fig. 2), not dissimilar to ACP/TE docking in *Jatropha curcas* [29]. To trap transient ACP-TE interactions *in vitro*, we designed, synthesized, and implemented chemical probes inspired by the enzymatic activity of the TE (Figs. 3C, 3D). Activity-based probe **3** was attached to Cr-cACP to form *crypto*-Cr-cACP **5** (Fig. 3B) [35], and indeed, a positive functional interaction between CrTE and Cr-cACP triggered a site-specific crosslinking reaction, effectively trapping the algal ACP-TE complex (Fig. 4B). A crosslinked ACP-TE complex was observed between *crypto*-Cr-cACP and CrTE using both chloroacrylic pantetheine probe **2** and longer chain α-bromopalmitic pantetheine probe **3** (Fig. 3B), demonstrating a positive functional ACP-TE interaction (Fig. 4B). Incubation with longer chain α-bromopalmitic pantetheine probe **3** resulted in full conversion to an ACP-TE complex, whereas chloroacrylic pantetheine probe **2** left unreacted CrTE, demonstrating the specificity of the CrTE probe (Fig. 4B). We did not find any evidence that the racemic nature of our synthesized α-bromopalmitic pantetheine molecule (**3**) and its 6 carbon analogue (**6**) affects the crosslinking reaction, similar to previous studies [26], and there are ongoing efforts in our laboratory to study this further.

Although UcTE and ChTE appeared to orient in a similar conformation to Cr-cACP as wildtype CrTE in computational docking studies, no crosslinked complex was detected using all three activity-based probes (**2**, **3**, and **6**), indicating that vascular plant TEs did not functionally interact with Cr-cACP *in vitro* (Figs. 4D, S9). To validate the results of our crosslinking assay *in vivo*, UcTE and ChTE were engineered into the *C. reinhardtii* chloroplast (Figs. S11, S12). Although TEs were expressed at relatively high levels (Fig. S12) and enzymatic activity of the TEs was confirmed (Fig. 5), no significant change in fatty acid content or composition was detected in transgenic Cr strains expressing vascular plant TEs, confirming that UcTE and ChTE were unable to hydrolyze fatty acids attached to Cr-cACP due to a lack of molecular recognition.

To further demonstrate the specificity of ACP-TE interactions, the Cr mitochondrial ACP (Cr-mACP) did not display favorable binding to CrTE *in silico* (Fig. S4), as the model of docked Cr-mACP/CrTE predicted a greater distance between the ACP serine and TE cysteine than in the Cr-cACP/CrTE model as well as a different binding orientation that does not appear to facilitate acyl transfer (Fig. S5). This result was validated *in vitro*, as Cr-mACP did not form a crosslinked complex with CrTE (Fig. S10).

The 16 carbon α-bromopantetheinyl-ACP served as a much better substrate mimic than chloroacrylic pantetheinyl-ACP (Fig. 4B). Similar selectivity has been demonstrated with polyketide KS domains that accept elongated hydrocarbon products, indicative of enhanced substrate binding [25]. Since the TE only acts upon a mature fatty-acyl-ACP, we propose that in addition to protein-protein interactions, the acyl chain attached to the ACP is important in guiding the thioester moiety towards the TE active site cysteine residue. Our modeling studies reveal a shallow hydrophobic binding cleft close to the CrTE active site (Fig. 2C). Perhaps there is a synergistic effect at work, whereby the acyl chain helps orient the fatty acid substrate in the correct conformation for hydrolysis to occur.

To test whether the length of the acyl chain attached to the Cr-cACP factors into enzymatic hydrolysis by the CrTE, we synthesized a shorter chain derivative of **3** composed of only six carbons for activity-based crosslinking. Whereas C16 α-bromopalmitic pantetheine probe **3** resulted in full conversion to an ACP-TE species, its six carbon analogue **6** yielded significantly less Cr-cACP/CrTE complex formation (Fig. 4C). These results indicate that in addition to ACP-TE interactions, chain length plays a role in the hydrolytic process.

To further characterize the CrTE *in vivo* and test its specificity, transgenic *C. reinhardtii* overexpressing native CrTE was analyzed for fatty acid content and composition. Wildtype *C. reinhardtii* produces mostly 16:0 fatty acids and only negligible amounts of 14:0 and 18:1 [44]. The shift in fatty acid profile to increased levels of 14:0 by overexpressing CrTE is notable in that CrTE does not display high homology to plant FatB TEs, which have varied affinities for saturated fatty acyl-ACPs [10]. In fact, CrTE is more homologous to FatA TEs, which explains the slight increase in 18:1 (Fig. 6). The availability of acyl-ACP substrates for TE hydrolysis determines the fatty acid content observed. For example, when *Arabidopsis thaliana* FatB TE was expressed in *E. coli*, an organism which produces mostly 16:0, there was a significant increase in 14:0 [45]. Therefore, if CrTE has specificity for saturated acyl-ACPs, its overexpression most likely causes a stoichiometric imbalance of CrTE to Cr-cACP in the plastid, which results in premature hydrolysis of available acyl-ACPs and accumulation of shorter chain fatty acids. The observation that the pool of 16:0-ACPs was diminished in this strain compared to wildtype supports this hypothesis (Fig. 6).

In plants, it is postulated that medium chain FatB TEs and FatA TEs are descendents of an ancient, ubiquitous FatB TE specific for 16:0-ACP [10]. A dedicated 16:0-acyl-ACP TE has not been detected in *C. reinhardtii*, but activity-based crosslinking studies with the 16:0-acyl-ACP probe indicate that CrTE can hydrolyze 16:0-ACP substrates. Crosslinking with the short-chain derivative (**6**) shows that CrTE is less selective for shorter chain acyl-ACPs, but it is still able to process them. *In vivo* characterization illustrates that CrTE can act upon 14:0-ACPs and 16:0-ACPs, as well as 18:1-ACPs, suggesting promiscuity in hydrolytic activity by CrTE. Docking fatty acid substrates of varied chain lengths and degrees of unsaturation provides further evidence that CrTE can hydrolyze both saturated and 18:1 fatty acids (Fig. S3). We propose that CrTE is a unique TE with both FatA and FatB character.

Algal TEs remain largely uncharacterized, with the exception of a putative thioesterase (PtTE) from the diatom *Phaeodactylum tricornutum* [46]. PtTE is distinct from FatA and FatB TEs as well as prokaryotic TEs. However, overexpression of PtTE in *E. coli* increases the amount of monounsaturated fatty acids and total fatty acid content, similar to FatA TEs [46]. Upon sequencing of the *C. reinhardtii* genome [18], CrTE was initially annotated as a FatA TE based on homology to plant TEs. Yet, sequence alignment shows only dispersed regions of similarity with FatA TEs (Figs. 1, S1). Currently in the UniProt database, CrTE is designated a ‘Fat1’ TE [47]. Phylogenetic analysis reveals that CrTE exists in a separate clade from FatA and FatB TEs along with other algal TEs (Fig. S13). Perhaps the CrTE sits at the crossroads of thioesterase evolution, where it functioned as one of the first TEs to hydrolyze unsaturated fatty acids. This is the first evidence of a unique Fat1 TE with promiscuity and possibly high hydrolytic activity for saturated acyl-ACP substrates in algae.

Protein-protein interactions between the TE and ACP control the identity of the fatty acid end product, as illuminated by activity-based crosslinking. Here, we demonstrate for the first time that activity-based chemical probes can be used to trap FAS enzymes and study their interactions with endogenous biosynthetic machinery in algal chloroplasts. We have verified that *in vitro* activity-based crosslinking translates to expected phenotypes *in vivo*, as shown by engineering TEs into the Cr chloroplast and analyzing fatty acid content. Characterization of algal fatty acid biosynthetic enzymes has unveiled distinct features compared to established bacterial and plant type II FAS systems, exemplified by a unique TE detected in *C. reinhardtii*. Our results demonstrate that synergy between protein-protein interactions and substrate recognition mediates the hydrolysis of fatty acids by CrTE.

This work offers a new fundamental understanding of algal fatty acid synthase to guide future metabolic engineering. It is the first demonstration of protein-protein interactions used as a mechanism to control product identity. We successfully designed and implemented activity-based probes as a means to understand the molecular recognition between the algal ACP and algal/plant TEs. In addition, we interrogated the specificity of the TE using three different activity-based probes. This novel approach may be used to facilitate the engineering of fatty acid identity in algae and other organisms of interest for energy production.

## Materials and Methods

### Protein modeling and docking

For detailed methods we refer to the supporting information (Text S1). Proteins were modeled using Swissmodel [48] and docked using the Cluspro server [34]. The top 10 balanced models were manually examined using PyMol [49] and the distances between TE active site cysteine and ACP serine residues measured. Autodock was used to blindly dock fatty acid substrates onto the model of CrTE [50] (Figs. S2, S3, S4).

### Sequence alignment

Sequence-based alignments of ACPs and TEs were produced using TCoffee [51], and figures were created using ESPript [52]. Transit peptides were predicted using ChloroP1.1 [53] and TargetP1.1 [54]. Full TE and ACP sequence alignments are provided in Figs. S1 and S6.

### Protein expression and purification

Cr-mACP, Cr-cACP, CrTE, UcTE, and ChTE proteins were expressed in *E. coli* and purified according to standard protocols and TEs reduced prior to use. ACP hydrolase (ACPH) was used to form *apo*-Cr-cACP (Figs. 3A and S7), confirmed by MS analysis (Fig. S8). Thioesterase activity was confirmed kinetically by monitoring hydrolysis of para-nitrophenylhexanoate (Fig. 5) [40].

### Activity-based crosslinking as a determinant of functional binding

Crosslinking reactions contained phosphate buffer, pantetheine analogue (**2**, **3** or **6** in DMSO), MgCl_2_, ATP, ACP, CoA-A, CoA-D, CoA-E and Sfp [26]. After 1 h at 37°C, TE was added to the reaction mixture and further incubated at 37°C overnight. Anti-FLAG resin or Ni-NTA resin was added directly to the reaction mixtures at room temperature, centrifuged, washed, and proteins eluted with 1 M arginine (pH 3.5) or 100 mM imidazole. As a negative control, enzymes were incubated without pantetheine analogue.

### Engineering TEs into *C. reinhardtii* chloroplast

UcTE, ChTE, and CrTE genes were codon-optimized for the Cr chloroplast and cloned into plastid expression vectors (16). Microprojectile bombardment was used to transform *C. reinhardtii* with TE constructs [55]. PCR was used to screen for integration of TE genes into the chloroplast genome (Fig. S11) and expression of TEs was validated by Western blot analysis (Fig. S12) [42]. Plant and algal TE activity was confirmed kinetically by monitoring hydrolysis of para-nitrophenylhexanoate (Fig. 5) [40].

### Growth of transgenic *C. reinhardtii* strains and GC/MS analysis of fatty acid content

*Chlamydomonas reinhardtii* strains were grown on TAP media [56] agar plates supplemented with carbendazim, ampicillin and cefotaxime [57] under constant illumination. Transgenic strains were kept under kanamycin selection. Flasks containing TAP media were inoculated with single colonies and grown under constant shaking in a greenhouse. After 3 days, cultures were centrifuged and the cell pellet resuspended in methanolic acid. The suspension was incubated for 30 min at 65°C and fatty acid methyl esters extracted using hexanes. Fatty acid composition was determined by GC/MS analysis (Fig. 6). Statistical analyses were performed using SPSS (v13.0), and for all data analysis, a *p*-value<0.5 was considered statistically significant.

## Supporting Information

Figure S1 **Sequence alignment of FatA and FatB TEs with CrTE.** Structure-based sequence alignment of FatA TEs from *Arabidopsis thaliana* (AtTE_A), *Brassica napus* (BnTE_A), *Chlamydomonas reinhardtii* TE (CrTE) and FatB TEs from *Cuphea hookeriana* (ChTE_B1) and (ChTE_B2) and *Umbellularia californica* (UcTE_B). Conserved residues are highlighted in red and similar residues appear in blue boxes. The end of transit peptide sequences are indicated by a black arrow. Cys-Asn-His catalytic triad is indicated by an asterix under each residue. Symbols are denoted as: α, α-helices; β, β-strands; η, 3_10_ helices; TTT, strict α-turn; TT, strict β-turn. Sequence-based alignments were produced using TCoffee [S1]. The figure was created using ESPript [S2].
(TIF)

Figure S2 **Docking of fatty acid PPTs to CrTE.** Autodock4 was used to blindly dock various fatty acid-PPTs to CrTE. (*A*) C16:0-PPT, (*B*) C16:4-PPT, (*C*) C18:0-PPT, (*D*) C18:1-PPT and (*E*) C18:3-PPT. Left: cartoon representation of CrTE (grey) and top 10 models of substrate as sticks. Right: surface representation of CrTE (grey) and top 10 models of substrate as sticks. [PPT: phosphopantetheine].
(TIFF)

Figure S3 **Ensemble representation of protein-protein docking of Cr-cACP with (** ***A*** **) UcTE, (** ***B*** **) ChTE and (** ***C*** **) CrTE.**
(TIF)

Figure S4 **Structural docking simulation of Cr-mACP to CrTE, UcTE, and ChTE.** Docking of *C. reinhardtii* mitochondrial ACP (Cr-mACP) to thioesterases showing less favorable docking characteristics (<50% of the ACP models docks to the tentative active site of the TEs). (*A*) UcTE (*B*) ChTE (*C*) CrTE.
(TIF)

Figure S5 **Electrostatic potential maps of modeled Cr-cACP/CrTE and Cr-mACP/CrTE complexes.** (*A*) Cr-cACP (green) docked to CrTE (grey) showing a 13.5 Å distance between conserved serine on Cr-cACP and active site cysteine of CrTE; (*B*) Cr-mACP (orange) docked to CrTE (grey) showing a 18.8 Å distance between conserved serine on Cr-mACP and active site cysteine of CrTE; (*C*) Cr-cACP (electrostatic surface display) docked to CrTE (ribbon, grey); (*D*) Cr-mACP (electrostatic surface display) docked to CrTE (ribbon, grey); (*E*) Cr-cACP (ribbon, green) docked to CrTE (electrostatic surface display); (*F*) Cr-mACP (ribbon, orange) docked to CrTE (electrostatic surface display).
(TIFF)

Figure S6 **Sequence alignment of ACPs.** Structure-based sequence alignment of ACPs from *Escherichia coli* (Ec), *Spinacia oleracea* mitochondrial ACP (So-mACP), *Spinacia oleracea* chloroplastic ACP (So-cACP), *Chlamydomonas reinhardtii* mitochondrial ACP (Cr-mACP), and *Chlamydomonas reinhardtii* chloroplastic ACP (Cr-cACP). Conserved serine is indicated by an asterix. The end of transit peptide sequences are indicated by a black arrow. Symbols are denoted as: α, α-helices; β, β-strands; η, 3_10_ helices; TTT, strict α-turn; TT, strict β-turn. Sequence-based alignments were produced using TCoffee [S1]. The figure was created using ESPript [S2].
(TIF)

Figure S7 **Apofication activity of ACP hydrolase on Cr-cACP.** L: Benchmark ladder. 1) Ni-NTA purified Cr-cACP (elution 1), 2) Ni-NTA purified Cr-cACP (elution 2), 3 and 4 are crude cell lysates of Cr-cACP expression, 5) apofication of Cr-cACP at t = 1 h, 6) t = 4 h, 7) t = 8 h, and 8) t = 24 h, showing the faster running *apo*-Cr-cACP being formed upon cleavage of the phosphopantetheine arm by ACP hydrolase. This was confirmed by mass spectrometry (Fig. S8) and testing the ability of *apo*-Cr-cACP to get loaded using fluorescent pantetheine analogue **1** (Fig. 3a). The bands at 24 kDa are presumably the disulfide bonded *holo*-Cr-cACP dimer, which can be reduced by the addition of DTT. Proteolysis (bands <10 kDa) can be prevented by addition of 0.5% sodium azide and 1 mM PMSF to the reaction mixture (data not shown). 9) Purified ACP hydrolase.
(TIF)

Figure S8 **Mass spectra of (** ***A*** **)** ***holo*** **-CrACP and (** ***B*** **)** ***apo*** **-CrACP (loss of phosphopantetheine).** ESI-MS were recorded in positive mode. ACP-hydrolase (ACPH) from *Pseudomonas aeruginosa* [S20] was used to convert *holo*-CrACP to *apo*-CrACP *in vitro*, which was confirmed by MS to verify the loss of the pantetheine moiety from ACP. The activity of *apo*-CrACP was validated using a one-pot chemoenzymatic method [S3], in which *apo*-CrACP was incubated with Sfp, CoA A, D, and E, ATP and fluorescent pantetheine analogue **1** (Fig. 3A).
(TIFF)

Figure S9 **Activity-based crosslinking of** ***C. reinhardtii*** **cACP and plant TEs.** (*A*) *crypto*-Cr-cACP was formed using CoA -A, -D, -E, Sfp, ATP, and chloroacrylic pantetheine analogue **2**, and tested for its ability to functionally interact with ChTE and UcTE. Reactions were purified using anti-FLAG resin, eluted with 1 M arginine (pH 3.5), and loaded onto an 8% SDS-PAGE gel. (*B*) *crypto*-Cr-mACP was formed using chloroacrylic pantetheine analogue **6** and **3** and tested for its ability to functionally interact with ChTE and UcTE. Reactions were purified by anti-FLAG resin and eluted with 1 M arginine (pH 3.5) and loaded onto an 8% SDS-PAGE gel. No crosslinked complex was observed between the Cr-cACP and either ChTE or UcTE using all three crosslinking probes, indicating a lack of functional interaction between the two domains. During 24 h incubation at 37°C, reduced plant TEs show spontaneous oxidation (bands at ∼100 kDa). L = Benchmark protein ladder; Cr = *C. reinhardtii*; Ch = *C. hookeriana*; Uc = *U. californica*. (+) denotes addition of pantetheine analogue **2** (*A*), **6** or **3** (*B*); (−) indicates negative control reactions in which pantetheine analogues were omitted. (*) indicates the position a crosslinked complex would be observed. (#) is a co-purifying contamination.
(TIFF)

Figure S10 **Fluorescent loading of** ***C. reinhardtii*** **mACP and crosslinking of Cr-mACP with TEs.** (*A*) *crypto*-Cr-mACP was formed using CoA -A, -D, -E, Sfp, ATP, and fluorescent pantetheine analogue **1** [S3], validating post translational modification of Cr-mACP. *E. coli* ACP was modified with **1** as a positive control. Reactions were loaded on an 8% SDS-PAGE gel and visualized at 365 nm; (*B*) *crypto*-Cr-mACP was formed from chloroacrylic pantetheine analogue **2** and tested for its ability to functionally interact with CrTE. No crosslinked complex was observed between the Cr-mACP and CrTE (right 2 lanes), indicating a lack of functional interaction between the two proteins. The *E. coli* ACP and KSII serve as a positive experimental control; a crosslinked complex is observed between the *E. coli* ACP and KSII (left 2 lanes). Reactions were visualized on a 12% SDS-PAGE gel stained with Coomassie; (*C*) The Cr-mACP fails to generate a crosslinked complex with CrTE (left 2 lanes) and UcTE (right 2 lanes), demonstrating a lack of protein-protein interactions between either the CrTE or UcTE and Cr-mACP, visualized on a Coomassie-stained 12% SDS-PAGE gel. L = Benchmark protein ladder; Ec = *E. coli*; Cr = *C. reinhardtii*; Uc = *U. californica*. (+) denotes addition of pantetheine analogue **1** or **2**; (−) negative control reactions in which pantetheine analogues were omitted.
(TIF)

Figure S11 **PCR screen to identify** ***Chlamydomonas reinhardtii*** **transformants harboring thioesterases.** Initial screening of primary Cr transformants was carried out using PCR to determine clones with proper insertion of exogenous genes into the Cr chloroplast genome, and rescreened to ensure genetic stability. (*A*) PCR analysis of genomic chloroplast DNA to determine degree of homoplasmy in Cr transformed with CrTE. (*B*) PCR on inserted nucleotide sequence of Cr transformed with CrTE. (*C*) Left: PCR on genomic chloroplast DNA to determine degree of homoplasmy in Cr transformed with UcTE, ChTE and CrTE. Right: PCR on inserted nucleotide sequence of Cr transformed with UcTE, ChTE and CrTE.
(TIF)

Figure S12 **Western blot analysis confirming expression of TEs in** ***C. reinhardtii*** **chloroplast.** To validate heterologous TE expression in the Cr chloroplast, Western blot analysis was conducted on transgenic Cr strains. Protein was purified via affinity purification using M2 anti-FLAG resin and Western blot was carried out using anti-FLAG antibody. Gel images show Western blot results for TEs overexpressed in *C. reinhardtii*. Each lane detects expression of a different TE in transgenic Cr chloroplasts: 1) CrTE, 2) UcTE, 3) CrTE [positive control] 4) wildtype strain 137c (mt+) [negative control], 5) ChTE. White numbers (left of gel) indicate molecular weight in kDa. Red letters above each lane denote TE detected: Cr = *C. reinhardtii*; Uc = *U. californica*; Ch = *C. hookeriana*; (+) overexpression of native CrTE; (−) wildtype Cr strain 137c.
(TIFF)

Figure S13 **Thioesterase phylogeny.** The phylogenetic relationship between FatA, FatB, and Fat1 TEs was analyzed using a web-based phylogenetic analysis tool [S4]. (*A*) Phylogram of the relationship between FatA, FatB, and Fat1 TEs, generated by Phylogeny.fr [S4]; (*B*) Radial representation of the phylogram shown in A.
(TIFF)

Table S1 **Strains, plasmids, and restriction sites.**
(DOC)

Table S2 ***Chlamydomonas reinhardtii*** **strains.**
(DOC)

Table S3 ***C. reinhardtii*** **primers.**
(DOC)

Text S1 **Textual description of supporting materials and methods.**
(DOC)
