# Supplementary figures and images for: Manipulating Fatty Acid Biosynthesis in Microalgae for Biofuel through Protein-Protein Interactions
Source: PLoS One. 2012 Sep 13;7(9):e42949. doi: 10.1371/journal.pone.0042949 (PMC3441505; doi:10.1371/journal.pone.0042949)

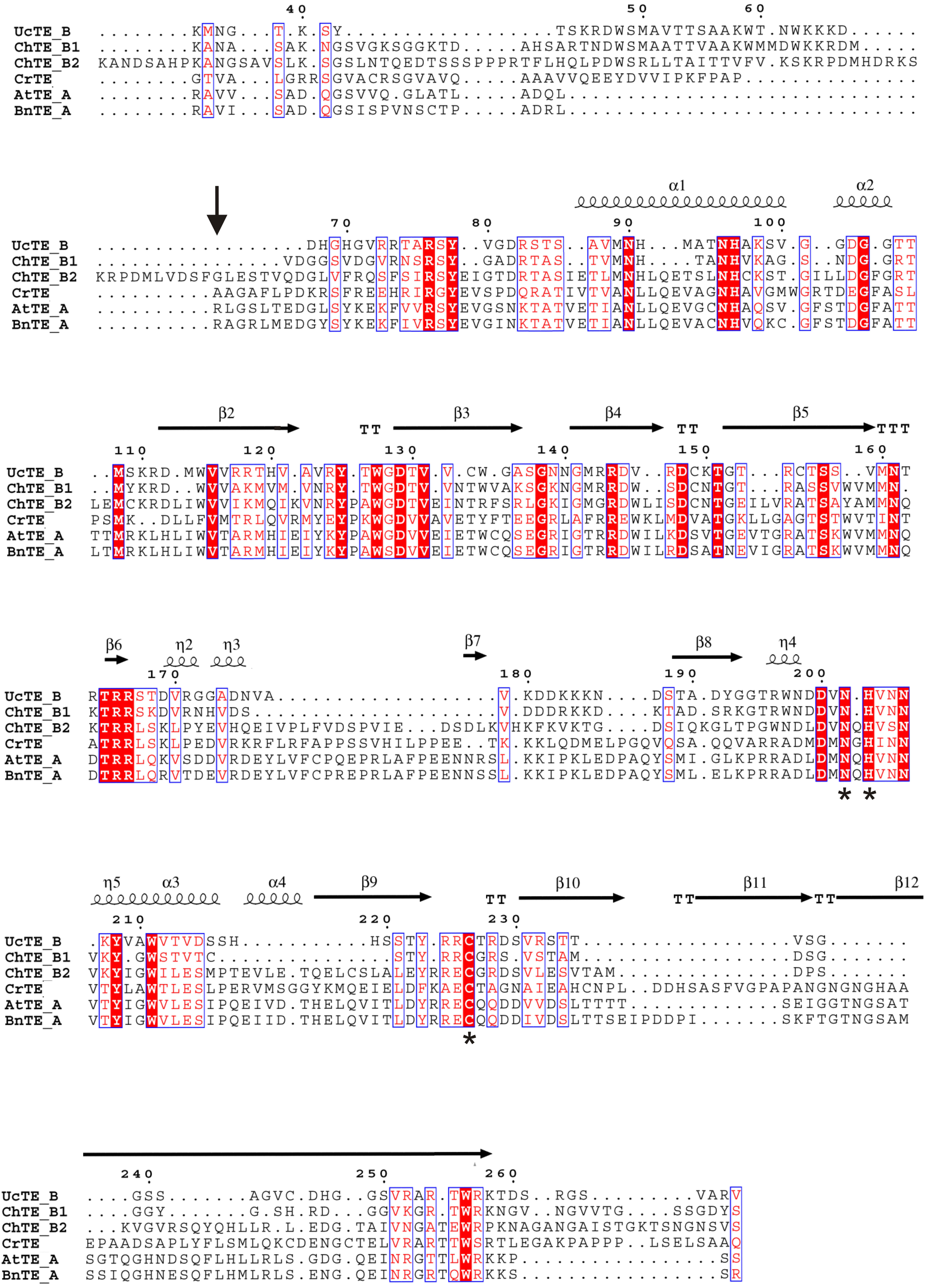

Supplement: Figure S1 — Sequence alignment of FatA and FatB TEs with CrTE. Structure-based sequence alignment of FatA TEs from Arabidopsis thaliana (AtTE_A), Brassica napus (BnTE_A), Chlamydomonas reinhardtii TE (CrTE) and FatB TEs from Cuphea hookeriana (ChTE_B1) and (ChTE_B2) and Umbellularia californica (UcTE_B). Conserved residues are highlighted in red and similar residues appear in blue boxes. The end of transit peptide sequences are indicated by a black arrow. Cys-Asn-His catalytic triad is indicated by an asterix under each residue. Symbols are denoted as: α, α-helices; β, β-strands; η, 310 helices; TTT, strict α-turn; TT, strict β-turn. Sequence-based alignments were produced using TCoffee [S1]. The figure was created using ESPript [S2]. (TIF) [file pone.0042949.s001.tif]

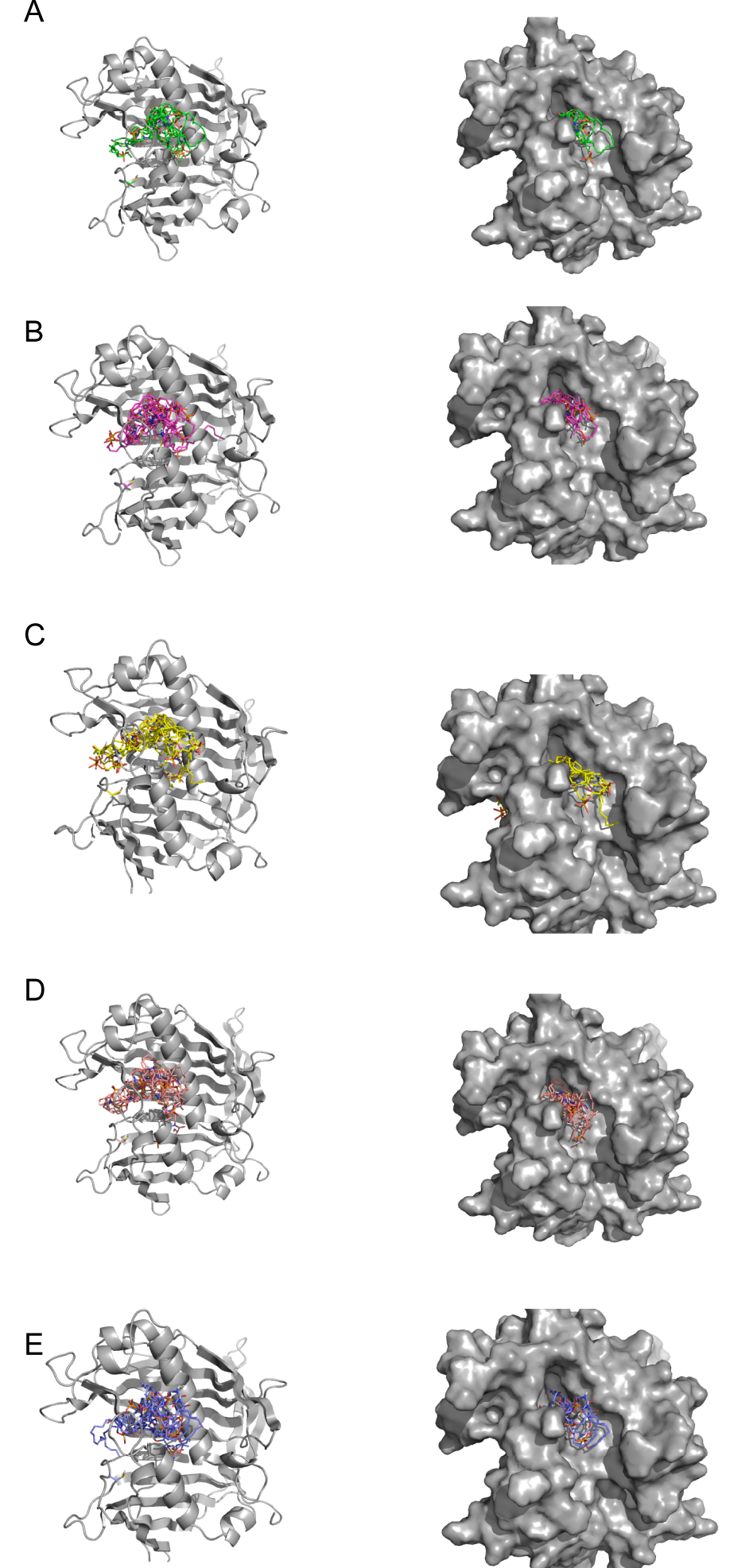

Supplement: Figure S2 — Docking of fatty acid PPTs to CrTE. Autodock4 was used to blindly dock various fatty acid-PPTs to CrTE. (A) C16:0-PPT, (B) C16:4-PPT, (C) C18:0-PPT, (D) C18:1-PPT and (E) C18:3-PPT. Left: cartoon representation of CrTE (grey) and top 10 models of substrate as sticks. Right: surface representation of CrTE (grey) and top 10 models of substrate as sticks. [PPT: phosphopantetheine]. (TIFF) [file pone.0042949.s002.tif]

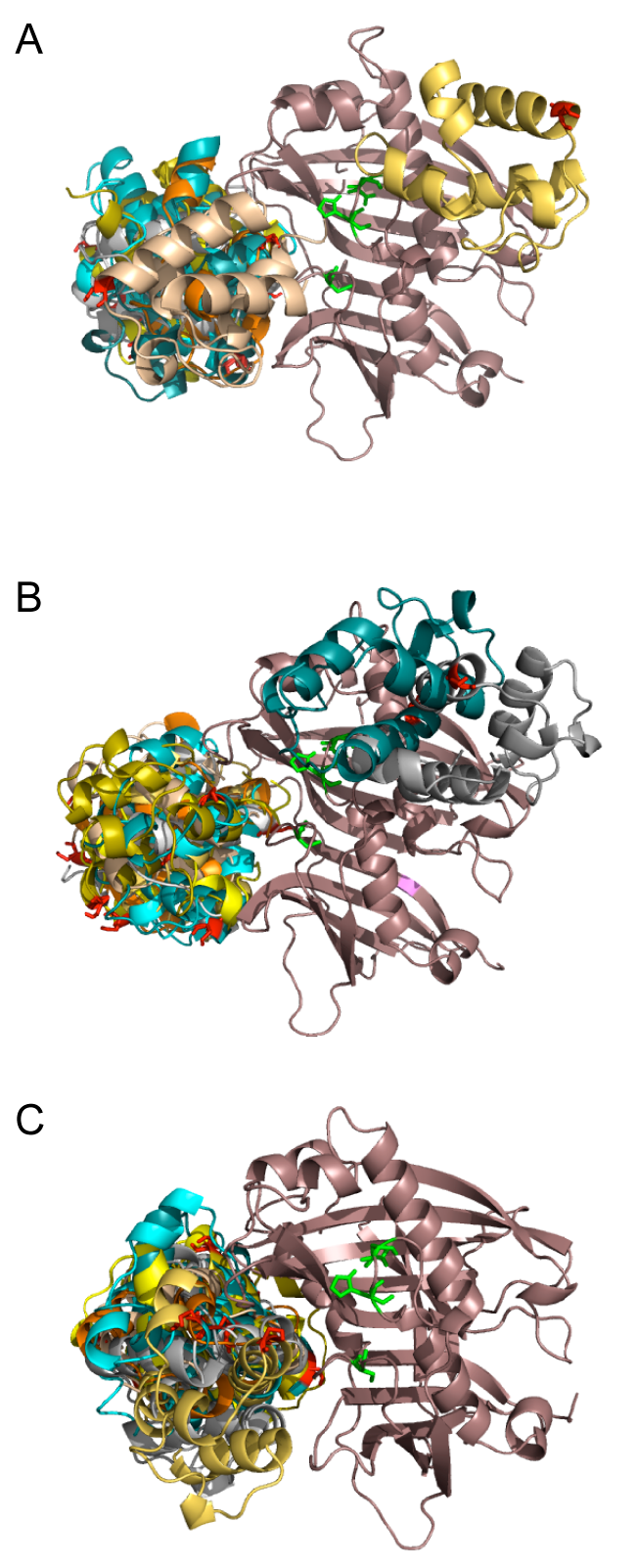

Supplement: Figure S3 — Ensemble representation of protein-protein docking of Cr-cACP with ( A ) UcTE, ( B ) ChTE and ( C ) CrTE. (TIF) [file pone.0042949.s003.tif]

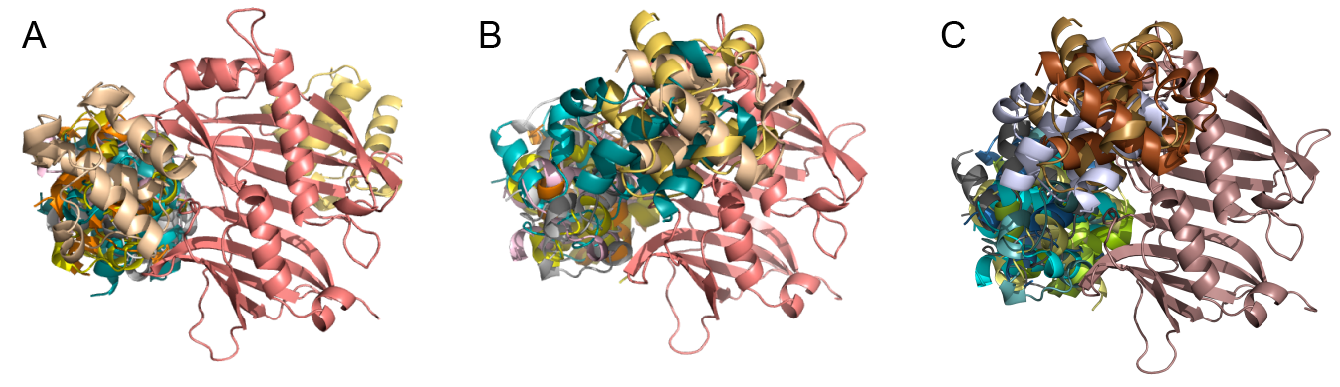

Supplement: Figure S4 — Structural docking simulation of Cr-mACP to CrTE, UcTE, and ChTE. Docking of C. reinhardtii mitochondrial ACP (Cr-mACP) to thioesterases showing less favorable docking characteristics (<50% of the ACP models docks to the tentative active site of the TEs). (A) UcTE (B) ChTE (C) CrTE. (TIF) [file pone.0042949.s004.tif]

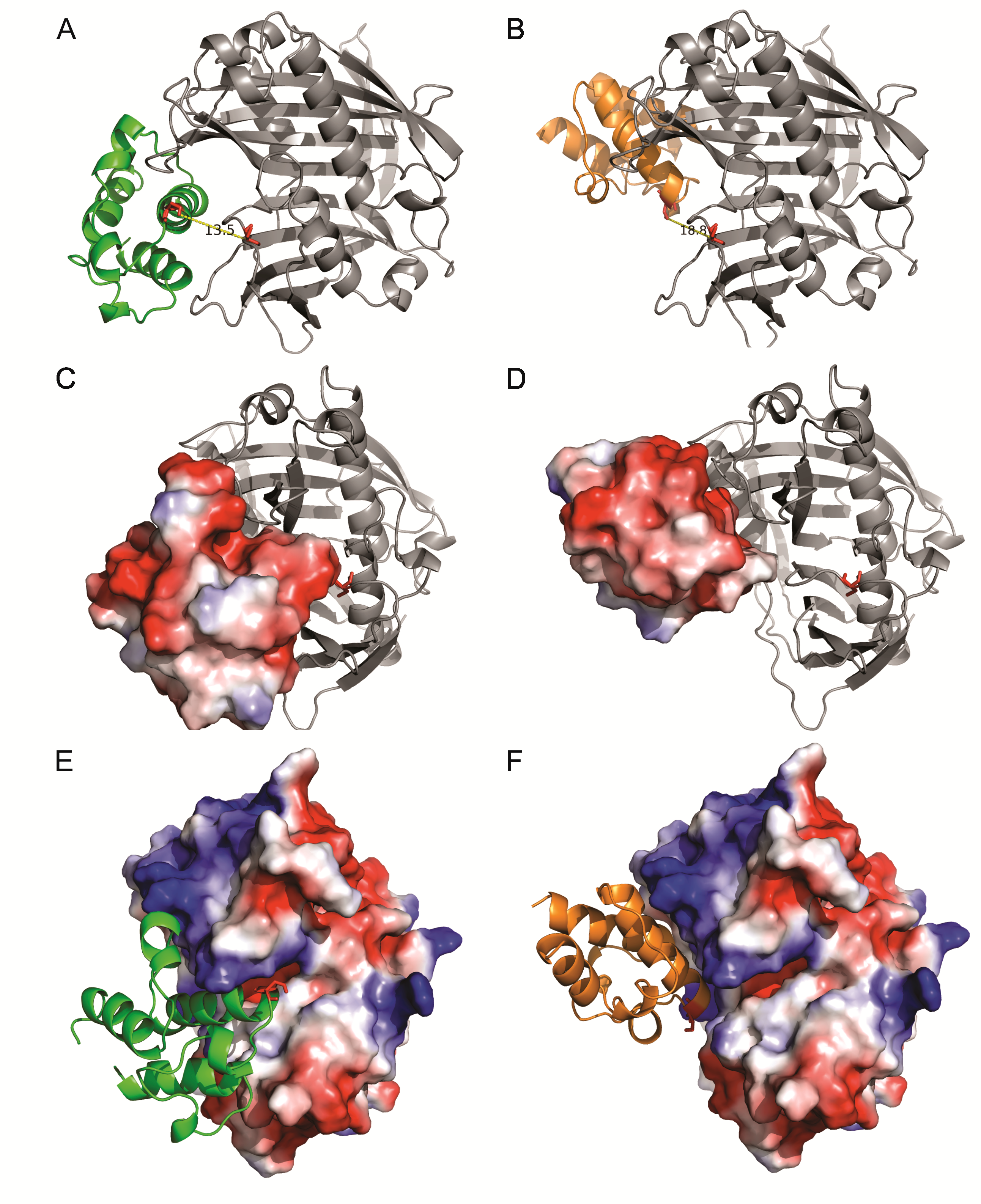

Supplement: Figure S5 — Electrostatic potential maps of modeled Cr-cACP/CrTE and Cr-mACP/CrTE complexes. (A) Cr-cACP (green) docked to CrTE (grey) showing a 13.5 Å distance between conserved serine on Cr-cACP and active site cysteine of CrTE; (B) Cr-mACP (orange) docked to CrTE (grey) showing a 18.8 Å distance between conserved serine on Cr-mACP and active site cysteine of CrTE; (C) Cr-cACP (electrostatic surface display) docked to CrTE (ribbon, grey); (D) Cr-mACP (electrostatic surface display) docked to CrTE (ribbon, grey); (E) Cr-cACP (ribbon, green) docked to CrTE (electrostatic surface display); (F) Cr-mACP (ribbon, orange) docked to CrTE (electrostatic surface display). (TIFF) [file pone.0042949.s005.tif]

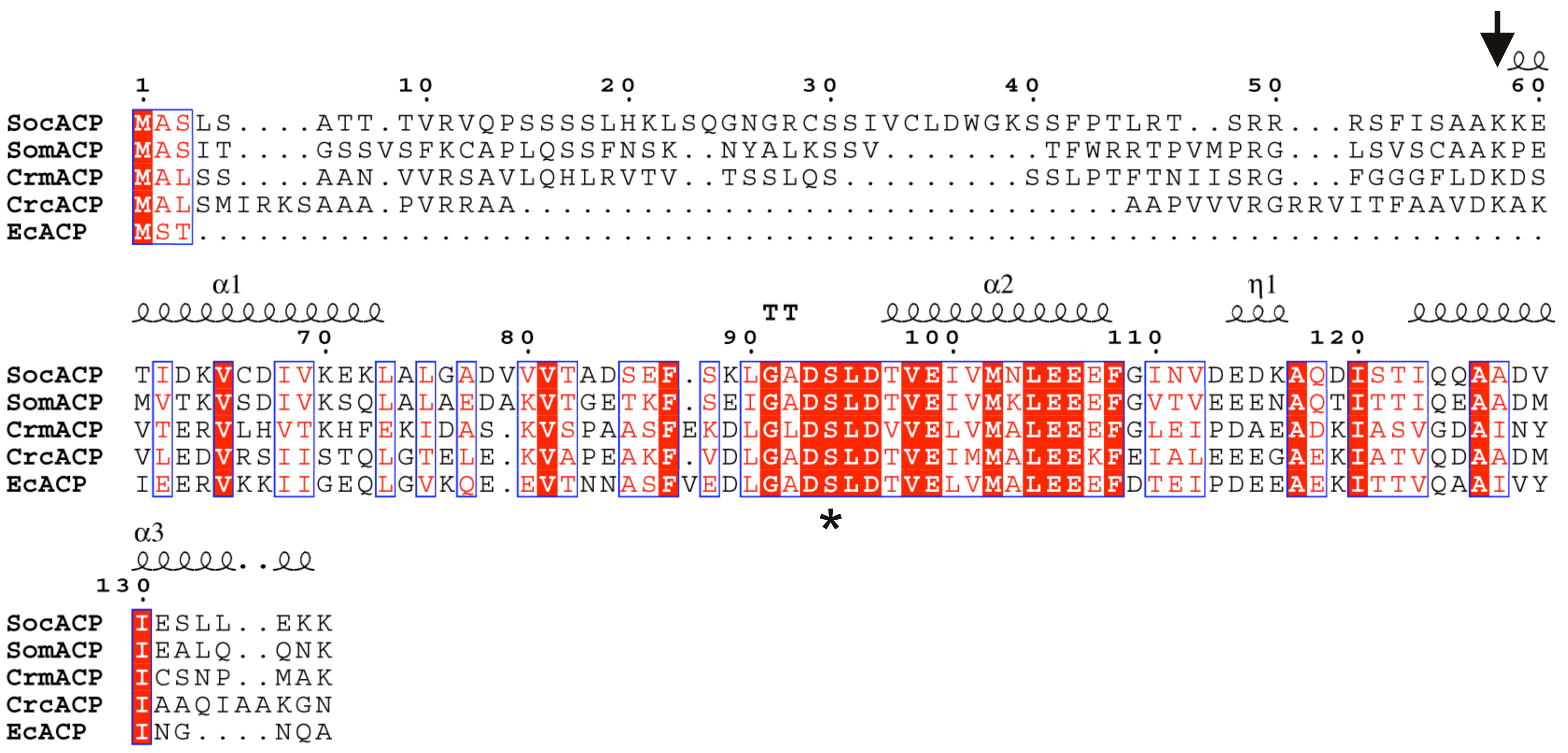

Supplement: Figure S6 — Sequence alignment of ACPs. Structure-based sequence alignment of ACPs from Escherichia coli (Ec), Spinacia oleracea mitochondrial ACP (So-mACP), Spinacia oleracea chloroplastic ACP (So-cACP), Chlamydomonas reinhardtii mitochondrial ACP (Cr-mACP), and Chlamydomonas reinhardtii chloroplastic ACP (Cr-cACP). Conserved serine is indicated by an asterix. The end of transit peptide sequences are indicated by a black arrow. Symbols are denoted as: α, α-helices; β, β-strands; η, 310 helices; TTT, strict α-turn; TT, strict β-turn. Sequence-based alignments were produced using TCoffee [S1]. The figure was created using ESPript [S2]. (TIF) [file pone.0042949.s006.tif]

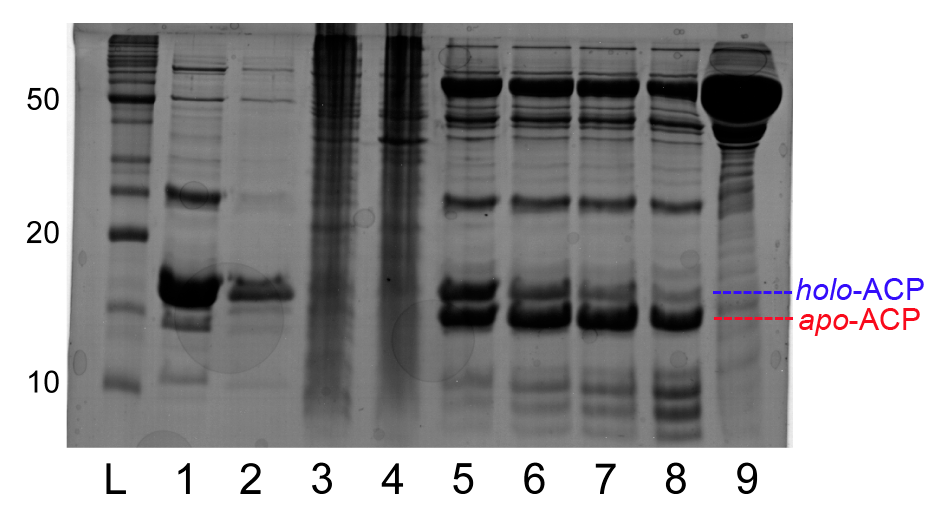

Supplement: Figure S7 — Apofication activity of ACP hydrolase on Cr-cACP. L: Benchmark ladder. 1) Ni-NTA purified Cr-cACP (elution 1), 2) Ni-NTA purified Cr-cACP (elution 2), 3 and 4 are crude cell lysates of Cr-cACP expression, 5) apofication of Cr-cACP at t = 1 h, 6) t = 4 h, 7) t = 8 h, and 8) t = 24 h, showing the faster running apo-Cr-cACP being formed upon cleavage of the phosphopantetheine arm by ACP hydrolase. This was confirmed by mass spectrometry (Fig. S8) and testing the ability of apo-Cr-cACP to get loaded using fluorescent pantetheine analogue 1 (Fig. 3a). The bands at 24 kDa are presumably the disulfide bonded holo-Cr-cACP dimer, which can be reduced by the addition of DTT. Proteolysis (bands <10 kDa) can be prevented by addition of 0.5% sodium azide and 1 mM PMSF to the reaction mixture (data not shown). 9) Purified ACP hydrolase. (TIF) [file pone.0042949.s007.tif]

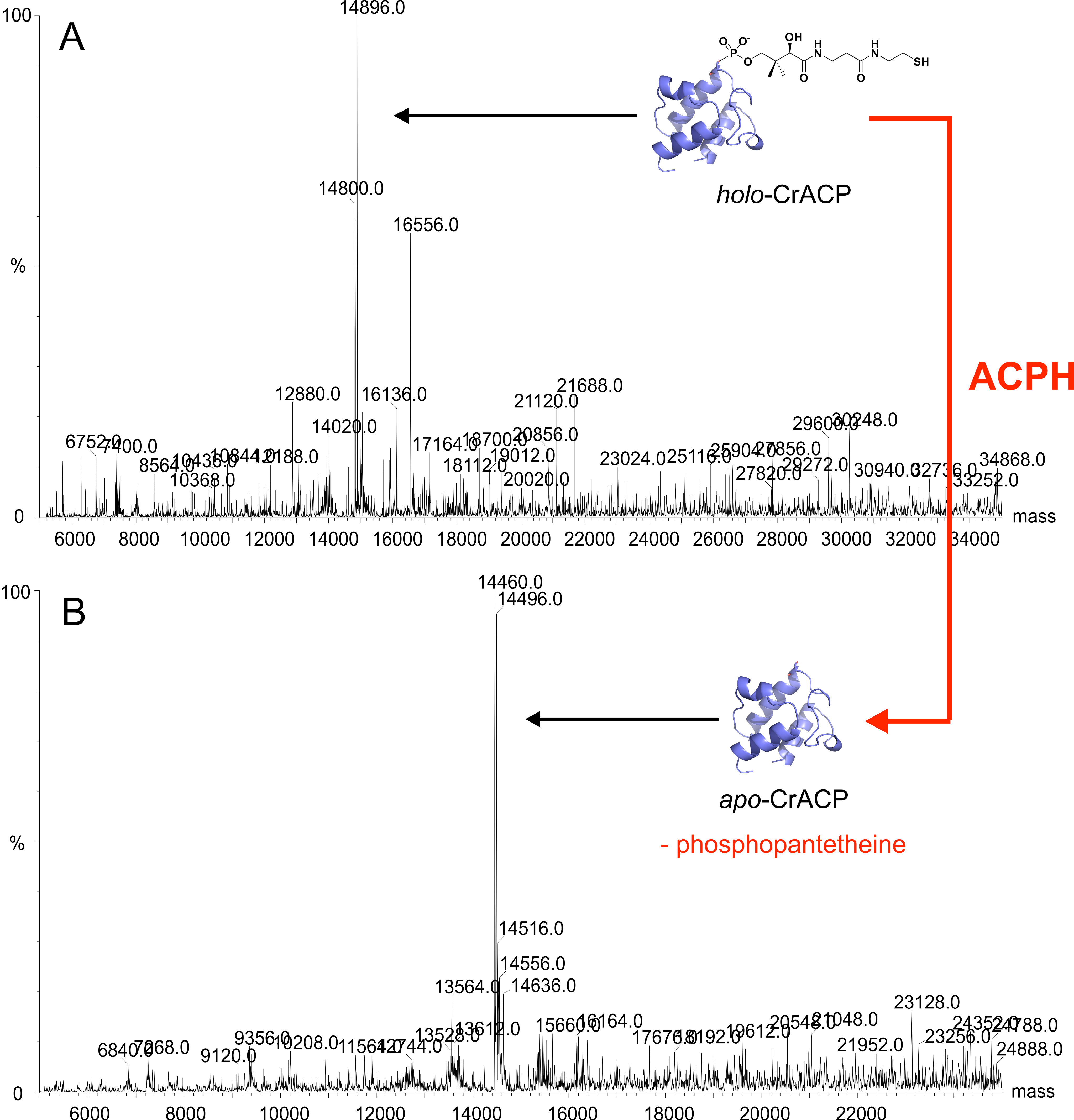

Supplement: Figure S8 — Mass spectra of ( A ) holo -CrACP and ( B ) apo -CrACP (loss of phosphopantetheine). ESI-MS were recorded in positive mode. ACP-hydrolase (ACPH) from Pseudomonas aeruginosa [S20] was used to convert holo-CrACP to apo-CrACP in vitro, which was confirmed by MS to verify the loss of the pantetheine moiety from ACP. The activity of apo-CrACP was validated using a one-pot chemoenzymatic method [S3], in which apo-CrACP was incubated with Sfp, CoA A, D, and E, ATP and fluorescent pantetheine analogue 1 (Fig. 3A). (TIFF) [file pone.0042949.s008.tif]

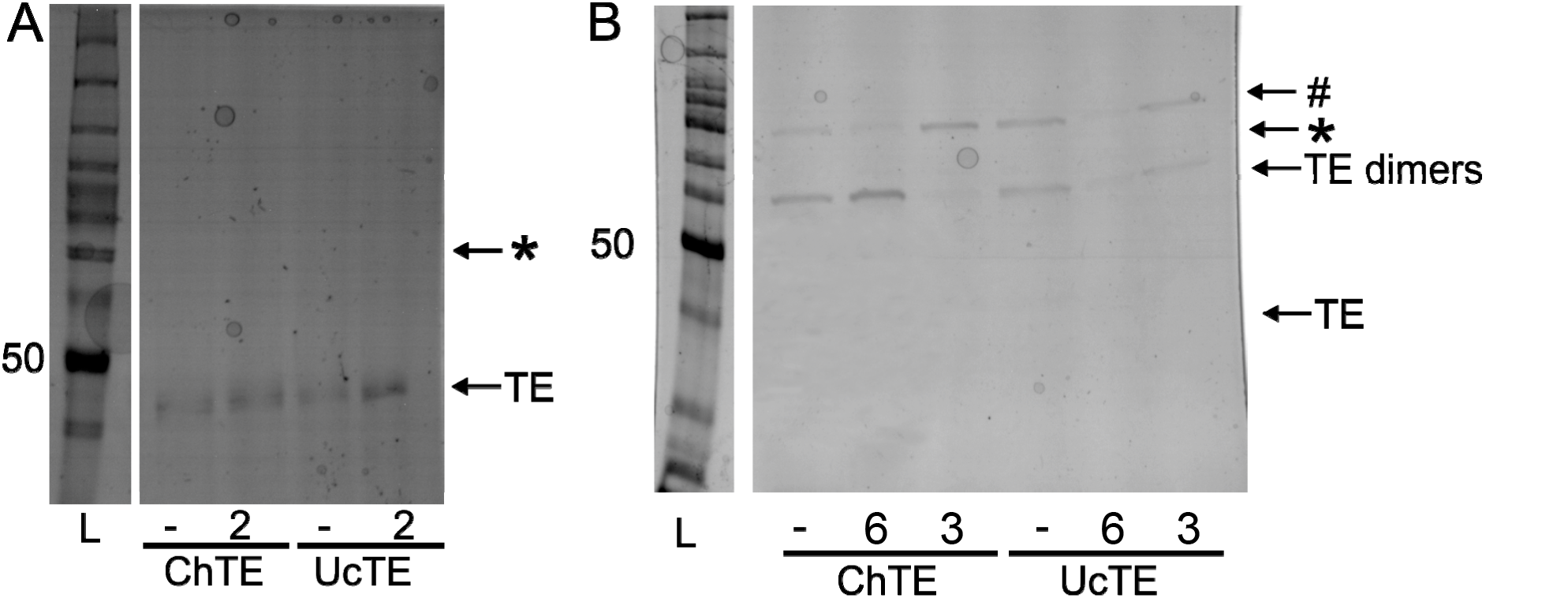

Supplement: Figure S9 — Activity-based crosslinking of C. reinhardtii cACP and plant TEs. (A) crypto-Cr-cACP was formed using CoA -A, -D, -E, Sfp, ATP, and chloroacrylic pantetheine analogue 2, and tested for its ability to functionally interact with ChTE and UcTE. Reactions were purified using anti-FLAG resin, eluted with 1 M arginine (pH 3.5), and loaded onto an 8% SDS-PAGE gel. (B) crypto-Cr-mACP was formed using chloroacrylic pantetheine analogue 6 and 3 and tested for its ability to functionally interact with ChTE and UcTE. Reactions were purified by anti-FLAG resin and eluted with 1 M arginine (pH 3.5) and loaded onto an 8% SDS-PAGE gel. No crosslinked complex was observed between the Cr-cACP and either ChTE or UcTE using all three crosslinking probes, indicating a lack of functional interaction between the two domains. During 24 h incubation at 37°C, reduced plant TEs show spontaneous oxidation (bands at ∼100 kDa). L = Benchmark protein ladder; Cr = C. reinhardtii; Ch = C. hookeriana; Uc = U. californica. (+) denotes addition of pantetheine analogue 2 (A), 6 or 3 (B); (−) indicates negative control reactions in which pantetheine analogues were omitted. (*) indicates the position a crosslinked complex would be observed. (#) is a co-purifying contamination. (TIFF) [file pone.0042949.s009.tif]

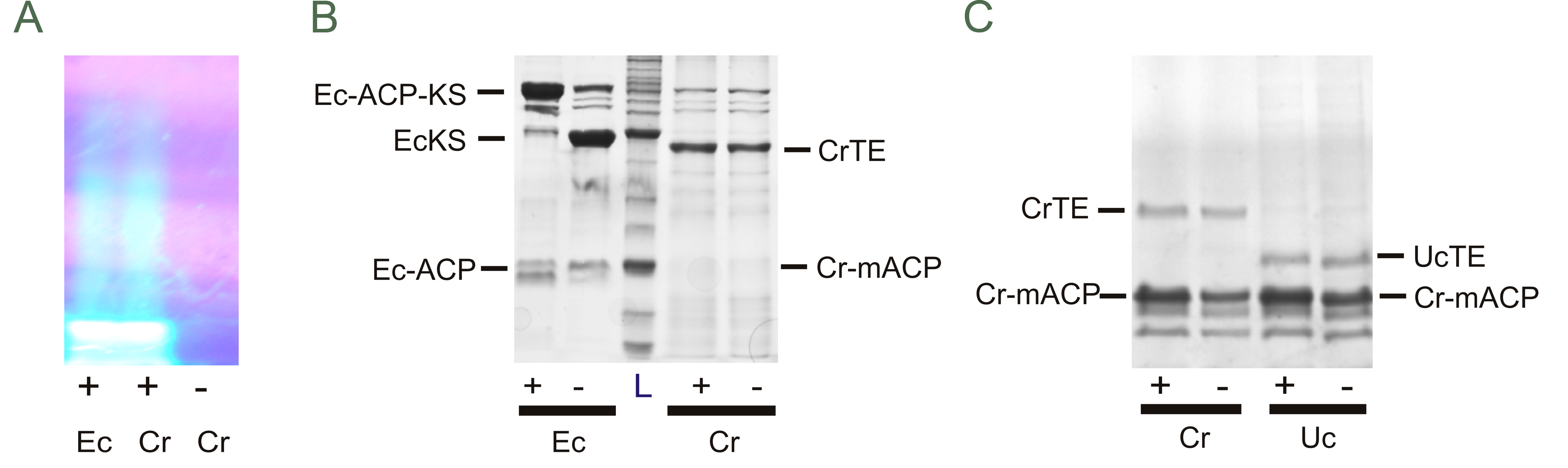

Supplement: Figure S10 — Fluorescent loading of C. reinhardtii mACP and crosslinking of Cr-mACP with TEs. (A) crypto-Cr-mACP was formed using CoA -A, -D, -E, Sfp, ATP, and fluorescent pantetheine analogue 1 [S3], validating post translational modification of Cr-mACP. E. coli ACP was modified with 1 as a positive control. Reactions were loaded on an 8% SDS-PAGE gel and visualized at 365 nm; (B) crypto-Cr-mACP was formed from chloroacrylic pantetheine analogue 2 and tested for its ability to functionally interact with CrTE. No crosslinked complex was observed between the Cr-mACP and CrTE (right 2 lanes), indicating a lack of functional interaction between the two proteins. The E. coli ACP and KSII serve as a positive experimental control; a crosslinked complex is observed between the E. coli ACP and KSII (left 2 lanes). Reactions were visualized on a 12% SDS-PAGE gel stained with Coomassie; (C) The Cr-mACP fails to generate a crosslinked complex with CrTE (left 2 lanes) and UcTE (right 2 lanes), demonstrating a lack of protein-protein interactions between either the CrTE or UcTE and Cr-mACP, visualized on a Coomassie-stained 12% SDS-PAGE gel. L = Benchmark protein ladder; Ec = E. coli; Cr = C. reinhardtii; Uc = U. californica. (+) denotes addition of pantetheine analogue 1 or 2; (−) negative control reactions in which pantetheine analogues were omitted. (TIF) [file pone.0042949.s010.tif]

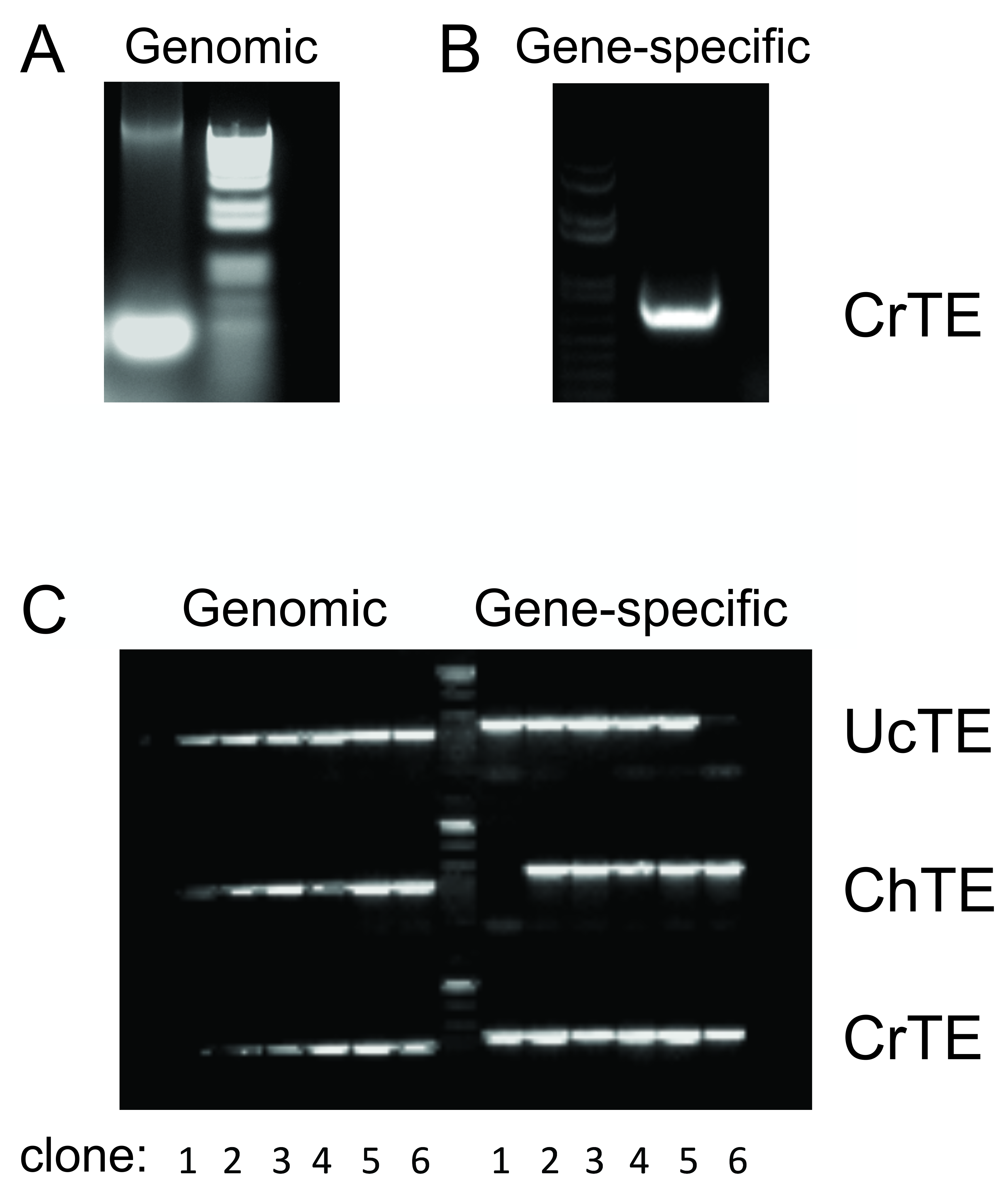

Supplement: Figure S11 — PCR screen to identify Chlamydomonas reinhardtii transformants harboring thioesterases. Initial screening of primary Cr transformants was carried out using PCR to determine clones with proper insertion of exogenous genes into the Cr chloroplast genome, and rescreened to ensure genetic stability. (A) PCR analysis of genomic chloroplast DNA to determine degree of homoplasmy in Cr transformed with CrTE. (B) PCR on inserted nucleotide sequence of Cr transformed with CrTE. (C) Left: PCR on genomic chloroplast DNA to determine degree of homoplasmy in Cr transformed with UcTE, ChTE and CrTE. Right: PCR on inserted nucleotide sequence of Cr transformed with UcTE, ChTE and CrTE. (TIF) [file pone.0042949.s011.tif]

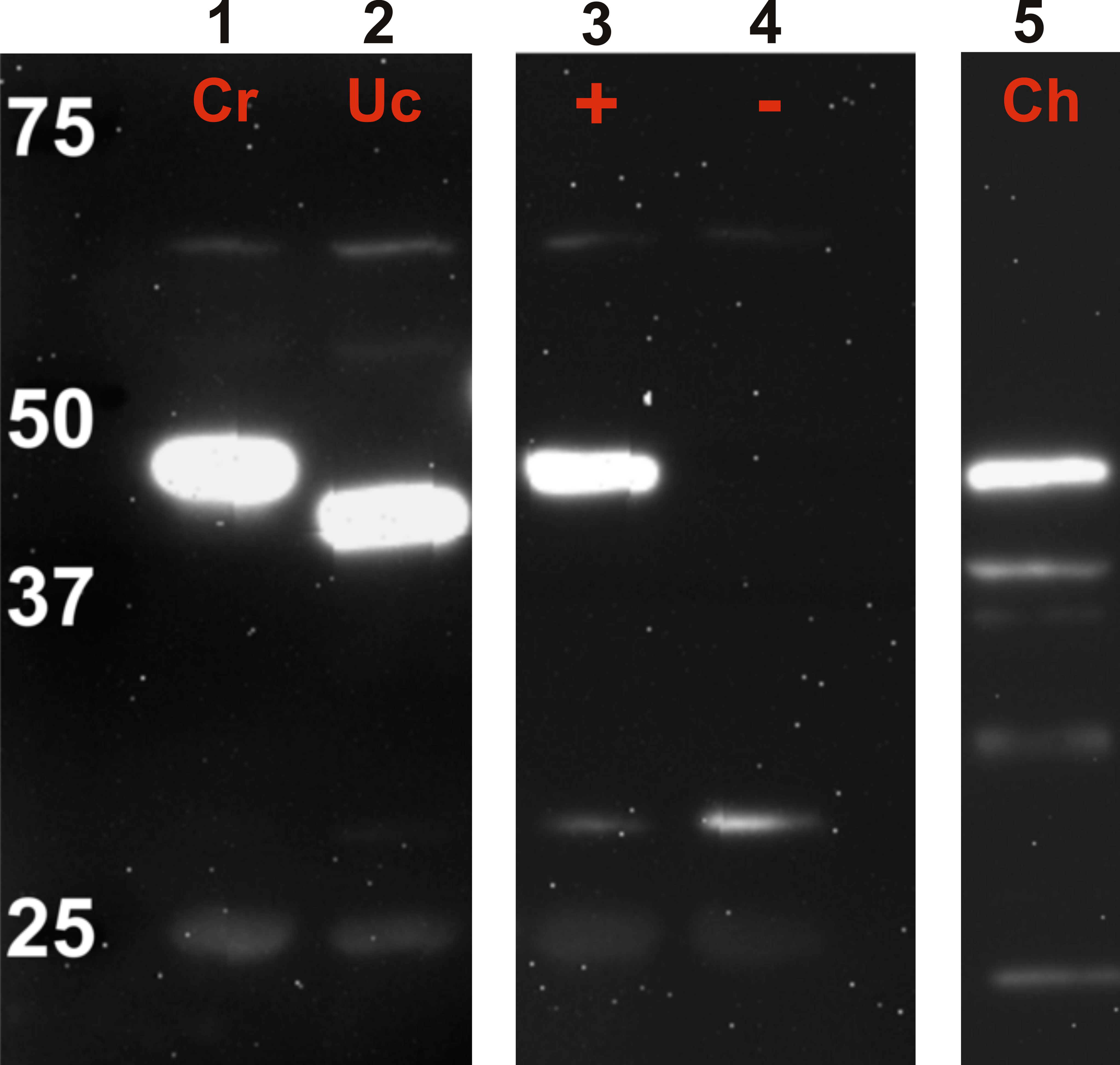

Supplement: Figure S12 — Western blot analysis confirming expression of TEs in C. reinhardtii chloroplast. To validate heterologous TE expression in the Cr chloroplast, Western blot analysis was conducted on transgenic Cr strains. Protein was purified via affinity purification using M2 anti-FLAG resin and Western blot was carried out using anti-FLAG antibody. Gel images show Western blot results for TEs overexpressed in C. reinhardtii. Each lane detects expression of a different TE in transgenic Cr chloroplasts: 1) CrTE, 2) UcTE, 3) CrTE [positive control] 4) wildtype strain 137c (mt+) [negative control], 5) ChTE. White numbers (left of gel) indicate molecular weight in kDa. Red letters above each lane denote TE detected: Cr = C. reinhardtii; Uc = U. californica; Ch = C. hookeriana; (+) overexpression of native CrTE; (−) wildtype Cr strain 137c. (TIFF) [file pone.0042949.s012.tif]

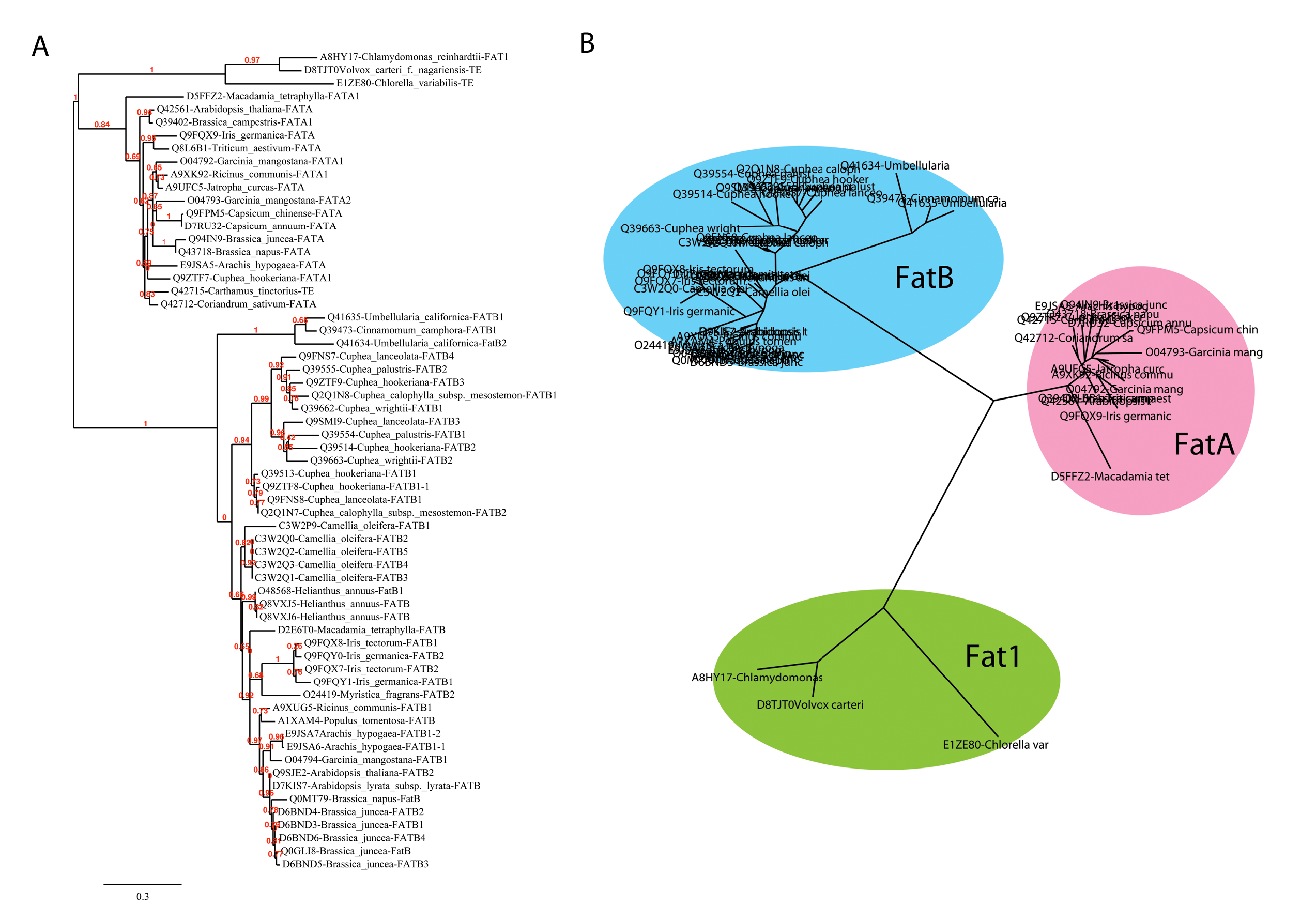

Supplement: Figure S13 — Thioesterase phylogeny. The phylogenetic relationship between FatA, FatB, and Fat1 TEs was analyzed using a web-based phylogenetic analysis tool [S4]. (A) Phylogram of the relationship between FatA, FatB, and Fat1 TEs, generated by Phylogeny.fr [S4]; (B) Radial representation of the phylogram shown in A. (TIFF) [file pone.0042949.s013.tif]
