# Supplementary material for: Manipulating Fatty Acid Biosynthesis in Microalgae for Biofuel through Protein-Protein Interactions
Source: PLoS One. 2012 Sep 13;7(9):e42949. doi: 10.1371/journal.pone.0042949 (PMC3441505; doi:10.1371/journal.pone.0042949)
Supplement: Table S1 — Strains, plasmids, and restriction sites. (DOC) [file pone.0042949.s014.doc]

Table S1. Strains, plasmids, and restriction sites

| **Enzyme** | **Vector** | **Organism** | **Restriction Sites** |
| --- | --- | --- | --- |
| Cr-cACP | pET-28b | *C. reinhardtii* | Nde1, Xho1 |
| Cr-mACP | pET-28b | *C. reinhardtii* | Nde1, Xho1 |
| CrTE | pET-21a | *C. reinhardtii* | Nde1, Xba1 |
| UcTE | pET-21a | *U. californica* | Nde1, Xba1 |
| ChTE | pET-21a | *C. hookeriana* | Nde1, Xba1 |
| ACPH | pET-24b | *P. aerigunosa* | Nde1, Xho1 |
