# Supplementary material for: Manipulating Fatty Acid Biosynthesis in Microalgae for Biofuel through Protein-Protein Interactions
Source: PLoS One. 2012 Sep 13;7(9):e42949. doi: 10.1371/journal.pone.0042949 (PMC3441505; doi:10.1371/journal.pone.0042949)
Supplement: Table S2 — Chlamydomonas reinhardtii strains. (DOC) [file pone.0042949.s015.doc]

**Table S2. *Chlamydomonas reinhardtii*** strains

| **Name** | **Type** | **Enzyme** | **Organism** | **Sequence** |
| --- | --- | --- | --- | --- |
| Cr_wt | Strain | Cr 137c (mt+) | *C. reinhardtii* | Wildtype strain [18] |
| Cr_CrTE | Strain | Cr 137c (mt+) CrTE | *C. reinhardtii* | Strain transformed with CrTE [19] |
| Cr_UcTE | Strain | Cr 137c (mt+) UcTE | *C. reinhardtii* | Strain transformed with UcTE [19] |
| Cr_ChTE | Strain | Cr 137c (mt+) ChTE | *C. reinhardtii* | Strain transformed with ChTE [19] |
