# Supplementary material for: Manipulating Fatty Acid Biosynthesis in Microalgae for Biofuel through Protein-Protein Interactions
Source: PLoS One. 2012 Sep 13;7(9):e42949. doi: 10.1371/journal.pone.0042949 (PMC3441505; doi:10.1371/journal.pone.0042949)
Supplement: Table S3 — C. reinhardtii primers. (DOC) [file pone.0042949.s016.doc]

**Table S3. *C. reinhardtii*** primers

| **Number** | **Type** | **Enzyme** | **Organism** | **Sequence** |
| --- | --- | --- | --- | --- |
| Primer 1 | Primer | 3’ rev for CrTE | Cr | ATTCCACCATCTGCTAATGCTT |
| Primer 2 | Primer | 3’ rev for UcTE | Uc | CATTCCAACGAGGAGTTAAAC |
| Primer 3 | Primer | 3’ rev for ChTE | Ch | ACTCCCGCGGTACTCAATGGTG |
| Primer 4 | Primer | 3HB fwd wt | Cr | CGCCACTGTCATCCTTTAAGT |
| Primer 5 | Primer | 3HB fwd | Cr | TGTTTGTTAAGGCTAGCTGC |
| Primer 6 | Primer | psbA 5’ UTR fwd | Cr | GTGCTAGGTAACTAACGTTTGATTTTT |
| Primer 7 | Primer | Control fwd homoplasty | Cr | CCGAACTGAGGTTGGGTTTA |
| Primer 8 | Primer | Control rev homoplasty | Cr | GGGGGAGCGAATAGGATTAG |
| Primer 9 | Primer | psbA 5’ UTR fwd | Cr | GGAAGGGGACGTAGGTACATAAA |
| Primer 10 | Primer | psbA 3’ rev | Cr | TTAGAACGTGTTTTGTTCCCAAT |
| Primer 11 | Primer | psbC 5’ UTR fwd | Cr | TGGTACAAGAGGATTTTTGTTGTT |
| Primer 12 | Primer | psbD 5’ UTR fwd | Cr | TGGTACAAGAGGATTTTTGTTGTT |
| Primer 13 | Primer | atpA 5’ UTR fwd | Cr | CCCCTTACGGGCAAGTAAAC |
