# Supplementary material for: Manipulating Fatty Acid Biosynthesis in Microalgae for Biofuel through Protein-Protein Interactions
Source: PLoS One. 2012 Sep 13;7(9):e42949. doi: 10.1371/journal.pone.0042949 (PMC3441505; doi:10.1371/journal.pone.0042949)
Supplement: Text S1 — Textual description of supporting materials and methods. (DOC) [file pone.0042949.s017.doc]

**SUPPORTING INFORMATION**

**Manipulating fatty acid biosynthesis in microalgae for biofuel through protein-protein interactions**

Jillian L. Blatti^a^, Joris Beld^a^, Craig A. Behnke^b^, Michael Mendez^b^, Stephen P. Mayfield^c^ and Michael D. Burkart^a^*

^a^ Department of Chemistry and Biochemistry, University of California, San Diego, 9500 Gilman Drive, La Jolla, CA 92093–0358, USA.

^b^ Sapphire Energy Inc., 3115 Merryfield Row, San Diego, CA 92121-1125. USA.

^c^ Division of Biological Sciences, University of California, San Diego, 9500 Gilman Drive, La Jolla, CA 92093–0368, USA.

*Corresponding author. Mailing address: University of California, San Diego, 9500 Gilman Drive, La Jolla, CA 92093–0358, USA. Phone: (858) 534–5673 or (858) 534–1551, E–mail: mburkart@ucsd.edu

biofuels | algae | fatty acid | acyl carrier protein | thioesterase |

**Materials and Methods**

**Materials**

All chemicals were purchased from Sigma-Aldrich, Fisher Science and VWR in biochemical grade. CoA -A, -D, -E and Sfp were expressed and purified using previously published methods [3]. Protein concentrations were determined using Bradford reagent. Buffers and media were prepared with ultrapure water. Anti-FLAG M2 affinity gel and anti-FLAG antibody were obtained from Sigma-Aldrich. Genomic DNA purification kit was purchased from Promega.

**Protein docking**

Protein sequences of Cr-cACP (ACP2, Q6UKY5_CHLRE), Cr-mACP (ACP1, Q6UKY4_CHLRE), CrTE (FAT1, A8HY17_CHLRE), ChTE (FATB2, Q39514_CUPHO) and UcTE (FATB1, Q41635) were obtained from the UniProt database. Sequence alignments were conducted using TCOFFEE [1] and ESPRIPT [2], based on the secondary structures of prokaryotic TEs (PDB: 2OWN, 2ESS) and ACPs (PDB: 2FAE). No structures of these algal and plant proteins are known, thus models were prepared using the automated Swissmodel server [5], which predicts protein structure based on homology modeling. The four steps used by Swissmodel are template superposition, target-template alignment, model building and energy minimization. In the latter, deviations in the protein structure, which have been introduced by the modeling algorithm when joining stiff fragments, are regularized by using the steepest descent energy minimization of the GROMOS96 force field. Models were compared with the models obtained from the i-Tasser server [6] and no significant deviations between the two homology modeling algorithms were observed. Cr-cACP (ACP2, 117 aa) was modeled after ACP from *Aquifex aeolicus* (PDB: 2EHTA, aa 40-114), which shows a sequence identity of 52.6%, a q-meanscore of 0.7 and a residual RMSD of 0.08. Cr-mACP (ACP1, 128 aa) models after malonyl-ACP from *Bacillus subtilis* (PDB: 2X2BA, aa 50-124) with a sequence identity of 52% and a q-meanscore of 0.3 and a residual RMSD of 0.09. While there are no algal or plant TE crystal structures available, there are two TE structures that show high homology and sequence identity to CrTE in the active site fold. CrTE (395 aa) was modeled after the TE from *Lactobacillus plantarum* (PDB: 2OWNA, aa 84-378), which shows a 17.6% overall sequence identity, 0.4 q-meanscore, and residual RMSD 0.47 Å, and the TE from *Bacteroides thetaiotaomicron* vpi-5482 (PDB: 2ESSA, aa 83-375, 17.5% overall sequence identity). PyMol structures from these two TEs were aligned with the CrTE homology model and residues in the catalytic fold were manually identified and aligned. The *L. plantarum* TE shows a 37.9% identity and the *B. thetaiotaomicron* TE shows a 41.4% sequence identity to CrTE around its active site Cys-His-Asn (8 Å).

With CrACP and CrTE homology models in hand, a panel of fourteen vascular plant TEs were identified that were homologous to CrTE and specific for short chain fatty acids. TEs included those from *Helianthus annuus* (FatB TE, 16:0 TE), *Umbellularia californica* (FatB1 TE), *Cinnamomum camphora* (FatB TE), *Cuphea hookeriana* (16:0 TE, FatB1 TE, FatB2 TE), *Jatropha curcas* (FatB1 TE, FatB2 TE, FatA TE), *Oryza sativa* (FatB TE), *Cuphea lanceolata* (FatB TE), *Cuphea wrightii* (FatB TE, 16:0 TE). Both UcTE (382 aa) and ChTE (415 aa) were also modeled after the TE from *Lactobacillus plantarum*, using the sequences of 99-367aa and 133-398aa, which have overall sequence identities of 18.2% and 22.6% and q-meanscore of 0.5 and 0.4, respectively.

Proteins were docked using the Cluspro server [7]. The top 10 balanced models were manually examined using PyMol [8], and the distances between thioesterase active site cysteine residues and acyl carrier protein serine residues measured.

Autodock was used to blindly dock fatty acid-phosphopantetheine (PPT) substrates onto the model of CrTE [9]. The protein structure was prepared by adding hydrogens using H++ [10] and removing water. The ligand was prepared by extracting the stearate-phosphopantetheine structure from the structure of spinach ACP (PDB: 2FVA) and the 18:0-phosphopantetheine was energy minimized using the PRODRG2 server [11]. Other fatty acid-phosphopantetheine substrates were energy minimized similarly. Autodock tools [12] were used to define torsional freedom. The cysteine active site residue was defined as a flexible residue, whereas the rest of the protein was considered rigid. A grid was deployed over the whole protein structure enabling the ligand to be docked in any location. A Lamarckian genetic algorithm and 2.5 Mi energy evaluations were used to dock the ligand onto the protein, using Autodock4. The obtained models were manually evaluated for relevance.

**Characterization of acyl carrier proteins of *C. reinhardtii***

Two sequences for *C. reinhardtii* fatty acid acyl carrier proteins exist in the NCBI and UniProt databases, ACP1 and ACP2. Based upon alignment with plant mitochondrial and chloroplastic ACPs, homology, and BLAST analysis, it is likely that ACP1 and ACP2 correspond to the mitochondrial and chloroplastic ACPs, respectively. Cr-mACP (ACP1) is a 128 amino acid protein with a molecular weight of 13.7 kDa and predicted pI of 4.80. Cr-cACP (ACP2), a 117 amino acid protein with a molecular weight of 12.4 kDa, has a predicted pI of 4.91. Sequence alignment of the Cr-cACP and Cr-mACP with ACPs from spinach (*Spinacia oleceria*) and bacteria (*Escherichia coli*) reveals a similar four-helix bundle, as shown in Fig. S6.

Cr-cACP contains a 54 amino acid transit peptide according to TargetP1.1 [13]. Interestingly, this algorithm predicts mitochondrial localization for Cr-cACP. ChloroP1.1 predicts a 15 residue plastid targeting sequence for Cr-cACP [14], whereas Mitoprot II v1.01 predicts a 38 amino acid cleavage site following a mitochondrial targeting sequence [15]. Predotar v1.03 also predicts mitochondrial localization for the Cr-cACP with slight possibility of localization to the chloroplast [16].

Cr-mACP contains a 64 amino acid mitochondrial transit peptide according to TargetP1.1 and mitochondrial localization [13]. A 23 residue chloroplast targeting peptide (cTP) is predicted for Cr-mACP by ChloroP1.1 [14], whereas Mitoprot II v1.01 predicts a 50 residue cleavage site following a mitochondrial targeting sequence [15]. Predotar v1.03 predicts chloroplastic localization for the Cr-mACP with slight possibility of localization to the mitochondria [16].

This ambiguity in predicting transit peptides of Cr-cACP and Cr-mACP suggests dual targeting of CrACPs to mitochondria and chloroplasts [17]. More experimental evidence is necessary in assigning either of the CrACPs a specific localization. It is clear from our crosslinking studies that the Cr-cACP and CrTE interact, giving confidence that the Cr-cACP is indeed the ACP involved in plastidial fatty acid biosynthesis.

Table S1. Strains, plasmids, and restriction sites

| **Enzyme** | **Vector** | **Organism** | **Restriction Sites** |
| --- | --- | --- | --- |
| Cr-cACP | pET-28b | *C. reinhardtii* | Nde1, Xho1 |
| Cr-mACP | pET-28b | *C. reinhardtii* | Nde1, Xho1 |
| CrTE | pET-21a | *C. reinhardtii* | Nde1, Xba1 |
| UcTE | pET-21a | *U. californica* | Nde1, Xba1 |
| ChTE | pET-21a | *C. hookeriana* | Nde1, Xba1 |
| ACPH | pET-24b | *P. aerigunosa* | Nde1, Xho1 |

**Cr-cACP, Cr-mACP, CrTE, ChTE and UcTE gene design**

Entelechon (Regensburg, Germany; www.entelechon.com) was used to backtranslate Cr-cACP and Cr-mACP into *E. coli* codon bias and genes were synthesized by GenScript (Piscataway, NJ). CrTE, UcTE and ChTE genes were codon optimized for *E. coli* and synthesized into a pET21a vector (DNA 2.0, Menlo Park, CA) in which a FLAG tag was added to the carboxy terminus.

**Cloning of Cr-cACP, Cr-mACP, CrTE, ChTE and UcTE**

Synthesized genes were subcloned into the multiple cloning site of pET28b (Cr-cACP and Cr-mACP) and pET21a (UcTE, CrTE, ChTE) vectors using Nde1 and Xho1 restriction sites and Nde1 and Xba1 restriction sites, respectively.

**Expression of Cr-cACP**

The Cr-cACP/pET28b plasmid was transformed into chemically competent BL21 (DE3) *E. coli* cells. 5 ml cultures in LB media, containing 50 μg/ml kanamycin, were grown from a single colony at 37 °C overnight. 1 liter cultures of LB media containing 50 mg/L kanamycin were inoculated with these 5 ml overnight cultures and grown for 4h at 37 °C until they reached an OD_600_ of 0.8, at which point they were induced with 500 μM IPTG (final concentration) and grown overnight at 25 °C. Cells were harvested by centrifugation at 4 °C for 30 minutes at 2000 rpm, and the obtained pellets were frozen at -80 °C.

**Expression of Cr-mACP**

The Cr-mACP/pET28b plasmid was transformed into chemically competent BL21 (DE3) *E. coli* competent cells. 5 ml LB cultures containing 50 μg/ml kanamycin were grown from a single colony at 37 °C overnight. 1 liter cultures of LB containing 50 mg/L kanamycin were inoculated with these 5 ml overnight cultures and grown for 4h at 37 °C. When they reached an OD_600_ of 0.8, cultures were induced with 1 mM IPTG (final concentration) and grown for 4h at 37 °C. Cells were harvested by centrifugation at 4 °C for 30 minutes at 2000 rpm, and the resultant pellets were frozen at -80 °C.

**Expression of TEs**

TE plasmids (pET21a/FLAG/CrTE, pET21a/FLAG/UcTE FatB1 and pET21a/FLAG/ChTE FatB2) were transformed into chemically competent BL21 (DE3) *E. coli* cells. 5 ml LB cultures containing 100 μg/ml ampicillin were grown from a single colony at 37 °C overnight. 1 liter cultures of LB containing 100 mg/L ampicillin were inoculated with these 5 ml overnight cultures and grown for 4h at 37 °C (OD_600_ of 0.8), induced with 500 μM IPTG (final concentration), and grown at 25 °C overnight. Cells were harvested by centrifugation at 4 °C for 30 minutes at 2000 rpm. Cell pellets were frozen at -80 °C.

**Expression of ACP hydrolase**

The *Pseudomonas aerigunosa* ACP hydrolase (ACPH) [20] in either pET24b (MBP-LVPRGSH-ACPH construct) or pMALc2x (MBP-ENLYFQ-HHHHHH-ACPH construct) plasmid was transformed into chemically competent BL21(DE3) *E. coli* cells and 5 ml LB cultures containing 50 μg/ml kanamycin or 100 μg/ml ampicillin were grown from a single colony at 37 °C overnight. 1 liter cultures of LB, in the presence of 50 mg/l kanamycin or 100 mg/l ampicillin, were inoculated with these 5 ml overnight cultures and grown for 4h at 37 °C. When they reached an OD_600_ of 0.8, cultures were induced with 500 μM IPTG (final concentration) and grown overnight at 25 °C. Cells were harvested by centrifugation at 4 °C for 30 minutes at 2000 rpm, and the obtained pellets frozen at -80 °C.

**Purification of *holo*-CrACP**

The pellet from a 1 liter culture was resuspended in 20 ml of lysis buffer (50 mM potassium phosphate pH 7.4, 500 mM sodium acetate, 0.1 mM EDTA, 20% glycerol) and lysed using a French pressure cell at 4 °C. Cell debris was spun down at 10,000 rpm at 4 °C for 1h. The His_6_-tagged protein was purified by Ni-NTA affinity chromatography. ACP was identified as *holo*-ACP by high resolution MS.

**Apofication of CrACP and purification of *apo*-CrACP**

The purified *holo*-CrACP (0.5 mg/ml) was transferred to dialysis tubing (3000 kDa MWCO) and ACPH, Tris-HCl (pH 8), NaCl, MgCl_2_, and MnCl_2_ added to final concentrations of 1 mg/ml and 50, 100, 15 and 1 mM, respectively. This mixture was dialyzed overnight at room temperature against 1 L of 50 mM Tris-HCl (pH 8), 100 mM NaCl, 15 mM MgCl_2_, and 1 mM MnCl_2_. The mixture containing the apofied CrACP was purified by Ni-NTA affinity chromatography, concentrated, and further purified by size-exclusion chromatography (FPLC). ACP was identified as *apo*-ACP by high resolution MS (Fig. S8).

**Purification of Cr-mACP**

The pellet from a 1 liter culture was suspended in 20 ml of lysis buffer (50 mM potassium phosphate pH 7.4, 500 mM sodium acetate, 0.1 mM EDTA, 20% glycerol) and lysed using a French pressure cell at 4 °C. Cell debris and inclusion bodies centrifuged at 10,000 rpm for 1h at 4 °C. The pellet was dissolved in 6 M guanidine hydrochloride and the His_6_-tagged protein was purified by Ni-NTA affinity chromatography under denaturing conditions. The unfolded protein was folded by sequential overnight dialysis in 6, 3, 2, 1, 0.5 M guanidine hydrochloride at 4 °C.

**Purification of TEs**

The pellet from 1 liter of culture was suspended in 20 ml of lysis buffer (50 mM potassium phosphate pH 7.4, 500 mM sodium acetate, 0.1 mM EDTA, 20% glycerol) and lysed using a French pressure cell at 4 °C. Cell debris was spun down at 10,000 rpm at 4 °C for 1h. The FLAG-tagged proteins were purified by FLAG-antibody affinity chromatography and eluted off the resin with 1 M arginine pH 3.5. Alternatively, the crude lysate was concentrated and purified using size-exclusion chromatography (FPLC).

**Reduction of TEs**

TEs were reduced with 10 mM DTT in TBS buffer (pH 7.4) and exhaustively dialyzed into PBS buffer (pH 6.5) prior to use.

**Synthesis of para-nitrophenylhexanoate**

Hexanoic acid (1g), N-hydroxysuccinimide (1 eq.) and Et_3_N (1.1 eq.) were dissolved in CH_2_Cl_2_, cooled to 0 °C and 1.1 eq. EDC slowly added while stirring. The mixture was allowed to warm to room temperature and stirred overnight. The activated ester was added dropwise to an ice-cold solution of 4-nitrophenol in CH_2_Cl_2_. After 3h at 0 °C and 5h at room temperature, ice was added and the mixture carefully acidified, extracted with ethyl acetate and the aqueous layer basified and extracted. The combined organic layers were dried and evaporated. The product was recrystallized form hot ethanol. (90%, LCMS-ESI calcd. (M+H) 237.2 found 237.2, ^1^H NMR (400 MHz, CDCl_3_) δ 0.90 (t, 3H), 1.3 (m, 2H), 1.5 (m, 2H), 2.3 (t, 2H), 7.55 (d, 2H), 8.23 (d, 2H)).

**Thioesterase activity assay**

*Chlamydomonas reinhardtii* thioesterase (CrTE), *Cuphea hookeriana* thioesterase (ChTE) and *Umbellularia californica* thioesterase (UcTE) were expressed in *E. coli* strain BL21 and purified by anti-FLAG resin, as described above. Separately, dense 100 ml TAP media cultures supplemented with the appropriate antibiotic of *C. reinhardtii* strains (chloroplastic) transformed with UCTE, ChTE and CrTE, were grown for 5 days, centrifuged, lysed via French pressure cell and the proteins purified by anti-FLAG affinity chromatography following the same procedure as for the enzymes expressed in *E. coli*. Protein concentrations were determined by Bradford assay, and the amounts used in the assay varied accordingly. Lipase and thioesterases were freshly reduced with dithiothreitol (pH 8) for 1 hour at room temperature and carefully buffer exchanged against 50 mM Tris-HCl pH 6.5 containing 5% glycerol. Thioesterase activity was determined by its ability to catalyze hydrolysis of 4-nitrophenylhexanoate. In a total volume of 150 μl, each reaction contained 10 mM Tris buffer (pH 7 or 8), 1.3% acetonitrile, 0.02% triton-X and 0.1 mg/ml enzyme. The mixtures of buffer, triton-X and enzymes were pre-incubated for 10 min at 37 °C and subsequently pre-warmed substrate added. Hydrolysis of para-nitrophenylhexanoate (1 mg/ml, dissolved in acetonitrile) at 30 °C was measured for 16 hours in duplicate on a Perkin Elmer HTS 7000 Bioassay reader at 340 nanometers.

**Thioesterase phylogeny**

The evolutionary origin of the algal/plant TE is unknown [21- 23]. A phylogenetic tree comprising various plant FatA TEs, FatB TEs, and the CrTE was created using web-based tools. All complete FatA, FatB and Fat1 protein sequences were obtained from the UniProt protein database and submitted for analysis using the one-click mode with default settings of Phylogeny.fr [4]. The results show that CrTE exists in a separate clade from FatA and FatB TEs phylogenetically, along with predicted TEs from other sequenced green algae, called ‘Fat1’ (Fig. S13). Crosslinking data suggests that CrTE is able to siphon 16:0-ACPs. Fatty acid analysis of *C. reinhardtii* overexpressing CrTE shows an increase in 18:1 fatty acids (Fig. 6). Thus, it is possible that CrTE can function as a FatA/B hybrid, able to hydrolyze both saturated and unsaturated fatty acids in the *C. reinhardtii* chloroplast.

**Purification of ACPH**

The pellet from a 1 liter culture was suspended in 20 ml of lysis buffer (50 mM potassium phosphate pH 7.4, 500 mM sodium acetate, 0.1 mM EDTA, 20% glycerol) and lysed using a French pressure cell at 4 °C. Cell debris was spun down at 10,000 rpm at 4 °C for 1h. The MBP-tagged protein was purified by amylose affinity chromatography and eluted from the resin with 100 mM maltose.

**One-pot loading of coumarin-pantetheine (1) onto ACP**

Typical one-pot loading reactions contain 50 mM phosphate buffer (pH 8), 2 mM coumarin-pantetheine (**1**) (in DMSO), 12.5 mM MgCl_2_, 8 mM ATP, 0.4 μg/ml ACP, 0.1 μg/ml CoA-A, 0.1 μg/ml CoA-D, 0.1 μg/ml CoA-E and 0.1 μg/ml Sfp [3]. This mixture (50 μl) was incubated for 1h at 37 °C to load fluorescent pantetheine **1** onto ACP. Proteins were separated on a 15% SDS-PAGE gel, and fluorescent *crypto*-ACP was visualized at 365 nm followed by Coomassie staining.

**Crosslinking experiments**

Crosslinking reactions contained 50 mM phosphate buffer (pH 8), 2 mM crosslinker (**2**, **3** or **6** in DMSO), 12.5 mM MgCl_2_, 8 mM ATP, 0.4 μg/ml ACP, 0.1 μg/ml CoA-A, 0.1 μg/ml CoA-D, 0.1 μg/ml CoA-E and 0.1 μg/ml Sfp. After 1h incubation at 37 °C, thioesterase (final concentration 0.1 μg/ml) was added and the reaction mixture further reacted. After overnight incubation, a 10 μl suspension of anti-FLAG resin (or Ni-NTA resin) was directly added to the mixtures, incubated for 30 minutes at room temperature, centrifuged, washed with sterile pH 7.4 TBS buffer, and the proteins eluted with 1 M arginine (pH 3.5) or 100 mM imidazole prior to being loaded onto 8% SDS-PAGE gels. Crosslinked complexes were confirmed by high resolution MS.

**Mass analysis of proteins by in gel digestion**

Crosslinked bands were excised from the gel, washed with H_2_O, 50/50 H_2_O/ACN, ACN and 100 mM ammonium bicarbonate prior to drying under vacuum (speed vacuum). Trypsin in a buffer containing CaCl_2_ and ammonium bicarbonate was added to the dried gel piece and the mixture incubated for 5h at 37 °C. The supernatant was dried under vacuum and 20 μl 5% formic acid added prior to MS analysis.

***Chlamydomonas reinhardtii* chloroplast engineering**

**Table S2. *Chlamydomonas reinhardtii*** strains

| **Name** | **Type** | **Enzyme** | **Organism** | **Sequence** |
| --- | --- | --- | --- | --- |
| Cr_wt | Strain | Cr 137c (mt+) | *C. reinhardtii* | Wildtype strain [18] |
| Cr_CrTE | Strain | Cr 137c (mt+) CrTE | *C. reinhardtii* | Strain transformed with CrTE [19] |
| Cr_UcTE | Strain | Cr 137c (mt+) UcTE | *C. reinhardtii* | Strain transformed with UcTE [19] |
| Cr_ChTE | Strain | Cr 137c (mt+) ChTE | *C. reinhardtii* | Strain transformed with ChTE [19] |

**Table S3. *C. reinhardtii*** primers

| **Number** | **Type** | **Enzyme** | **Organism** | **Sequence** |
| --- | --- | --- | --- | --- |
| Primer 1 | Primer | 3’ rev for CrTE | Cr | ATTCCACCATCTGCTAATGCTT |
| Primer 2 | Primer | 3’ rev for UcTE | Uc | CATTCCAACGAGGAGTTAAAC |
| Primer 3 | Primer | 3’ rev for ChTE | Ch | ACTCCCGCGGTACTCAATGGTG |
| Primer 4 | Primer | 3HB fwd wt | Cr | CGCCACTGTCATCCTTTAAGT |
| Primer 5 | Primer | 3HB fwd | Cr | TGTTTGTTAAGGCTAGCTGC |
| Primer 6 | Primer | psbA 5’ UTR fwd | Cr | GTGCTAGGTAACTAACGTTTGATTTTT |
| Primer 7 | Primer | Control fwd homoplasty | Cr | CCGAACTGAGGTTGGGTTTA |
| Primer 8 | Primer | Control rev homoplasty | Cr | GGGGGAGCGAATAGGATTAG |
| Primer 9 | Primer | psbA 5’ UTR fwd | Cr | GGAAGGGGACGTAGGTACATAAA |
| Primer 10 | Primer | psbA 3’ rev | Cr | TTAGAACGTGTTTTGTTCCCAAT |
| Primer 11 | Primer | psbC 5’ UTR fwd | Cr | TGGTACAAGAGGATTTTTGTTGTT |
| Primer 12 | Primer | psbD 5’ UTR fwd | Cr | TGGTACAAGAGGATTTTTGTTGTT |
| Primer 13 | Primer | atpA 5’ UTR fwd | Cr | CCCCTTACGGGCAAGTAAAC |

**Thioesterases plasmids construction for transformation into *Chlamydomonas reinhardtii***

All DNA and RNA manipulations were carried out using standard methods previously described [24]. Codon-optimized TE genes were cloned into a chloroplast expression vector, in which the insert is under the control of the 5’UTR and promoter sequence for the *psbD* gene and 3’UTR for the *psbA* gene. The kanamycin resistance gene (*aphA6*) is regulated by the 5’UTR and promoter sequence for *atpA* and 3’UTR for the *rbcL* gene. The transgene cassette is flanked by 5’ and 3’ homology regions which target the entire cassette to the 3HB locus of *Chlamydomonas reinhardtii.*

**Transformation of thioesterase plasmids into *Chlamydomonas reinhardtii 137c (mt+)***

*C. reinhardtii* cells were grown to late log phase (7 days) in TAP media in the presence of 0.5 mM 5-fluorodeoxyuridine at 23 °C, under constant illumination of 450 lux on a rotary shaker at 100 rpm. Cells (50 ml) were harvested by centrifugation (4000 x g, 23 °C, 5 min) and the supernatant was decanted and the cells resuspended in 4 ml TAP media prior to transformation by particle bombardment [25]. All transformations were performed under kanamycin (100 μg/ml) selection.

PCR was used to identify the transformed strains (Fig. S11). For PCR analysis, 10^6^ cells from plate or liquid culture were suspended in 10 mM EDTA and heated to 95 °C for 10 min and then cooled to 23 °C. This mixture was added to a typical PCR cocktail, containing MgCl_2_, dNTPs, PCR primer pairs, DNA polymerase and water. MgCl_2_ and EDTA concentrations were carefully controlled. Additionally, genomic DNA (extracted with a Promega Genomic DNA purification kit) was used as a template if crude algal lysates yielded unclear results.

To identify strains containing the thioesterase gene, a primer pair was used in which one anneals to a site in the 5’UTR and the other to the thioesterase coding segment. Positive clones yield the desired size PCR fragment. To determine the degree to which the endogenous gene locus has been displaced (heteroplasmic versus homoplasmic), a PCR reaction containing two sets of primers was used. The first pair amplifies the endogenous locus targeted by the expression vector. The second pair amplifies a constant, or control, region that is not targeted by the expression vector. The number of cycles was increased to >30 to enhance sensitivity. The most desired clones are those that yield a product from the constant region but not from the endogenous region.

To confirm expression of each thioesterase protein in transgenic *C. reinhardtii* strains, Western blot analysis was performed (Fig. S12). 5x10^8^ cells were collected by centrifugation from TAP media by centrifugation. Cells were resuspended in cold lysis buffer (25 mM Tris, 137 mM NaCl, 2.7 mM KCl, pH 7.4) supplemented with an EDTA-free protease inhibitor cocktail (Roche). Cells were lysed by sonication, followed by centrifugation. Proteins were purified by anti-FLAG affinity chromatography at 4 °C and the protein eluted off the resin using 0.1 M glycine pH 3.0. Proteins were separated by SDS-PAGE and transferred to PVDF membrane. The membrane was blocked with blocking solution for 30 min at room temperature, incubated with HRP-anti-FLAG antibody for 12h at 4 °C, washed, and visualized by HRP-substrate and chemiluminescent detection.

***Chlamydomonas* *reinhardtii* growth and fatty acid analysis**

*C.* *reinhardtii* strains were maintained on TAP media [26], supplemented with 40 μg/ml carbendazim, 500 μg/ml ampicillin and 100 μg/ml cefotaxime (TAP+C+A+C) [27] (and in the case of plant TEs, an additional 100 μg/ml kanamycin (+K)), agar plates under constant illumination. Sterile 150 ml flasks containing 50 ml TAP+C+A+C (+K) media were inoculated with single colonies and grown under constant shaking in a greenhouse. Cell density of the cultures was measured. After 3 days, cultures were centrifuged at 4000 rpm at 4 °C, and the cell pellet resuspended in 2 ml of a 1 M methanolic acid solution. The suspension was incubated for 30 min at 65 °C and the fatty acid methyl esters extracted using 2 ml of hexanes. The fatty acid composition was determined by GC/MS analysis on an Agilent 7890A GC system connected to a 5975C VL MSD quadrupole MS (EI). Helium was used as a carrier gas. A method employing a gradient of 110 °C to 200 °C at 15 °C /min followed by 20 minutes at 200 °C on a 60 meter DB23 column was used to accurately measure fatty acid methyl ester content and composition. Statistical analyses were performed using SPSS (v13.0), and for all data analysis, a *p*-value <0.5 was considered statistically significant.

**Activity-based pantetheine probes used in crosslinking studies**

Pantetheine analogue **2** was used in previous studies to elucidate ACP-KS interactions in prokaryotic systems [26]. Here, C16-α-bromoamide probe **3** was designed as a site-specific inhibitor of thioesterase activity, in which a reactive bromide was placed α to the site of nucleophilic attack by the TE. We hypothesized that the longer aliphatic tail of **3** could possibly help orient the amide towards the TE active site. To test TE specificity, a six carbon analogue (**6**) of **3** was also envisioned. Activity-based probes **3** and **6** were synthesized by adapting methods previously described [28].


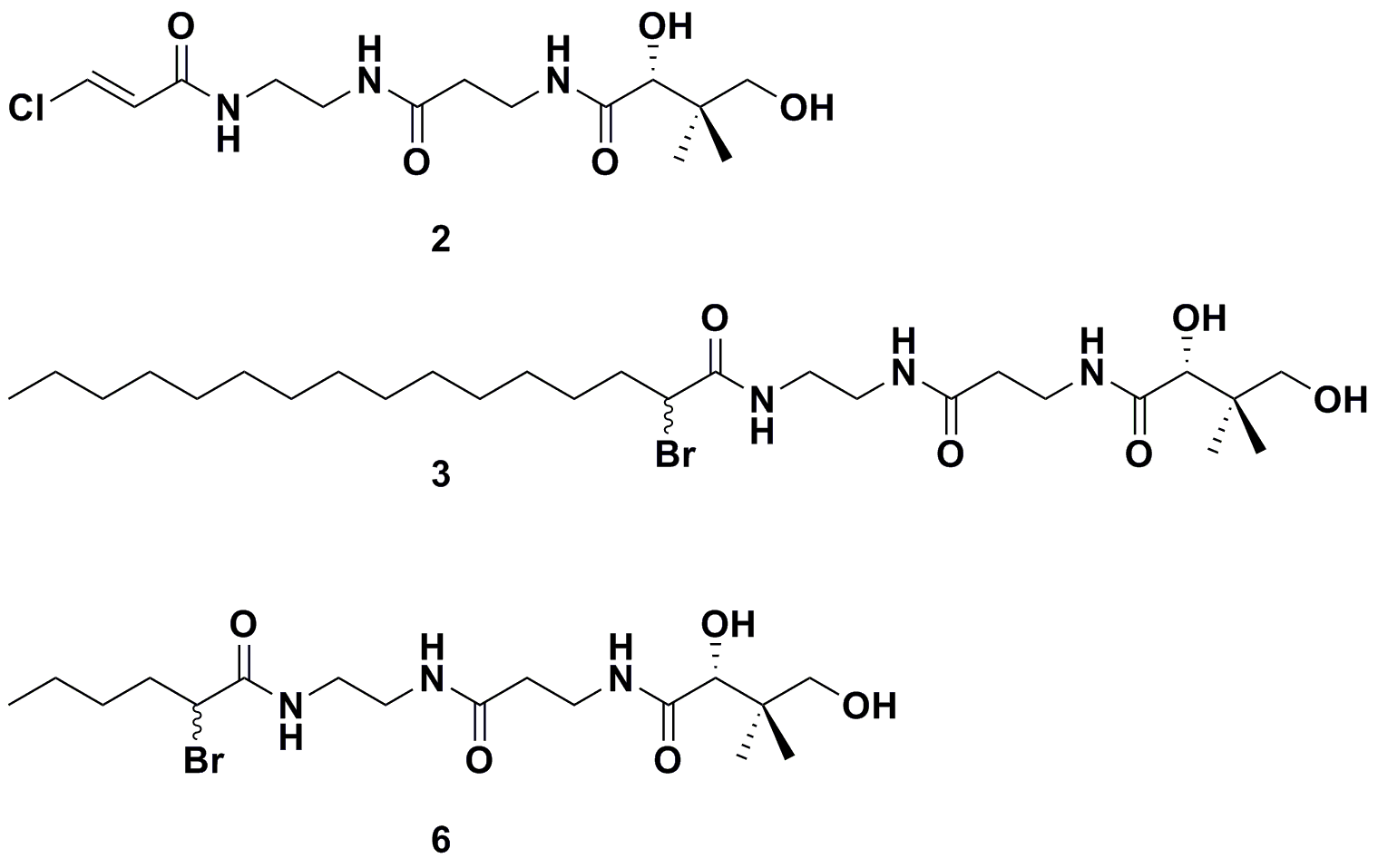


**Summary of synthesis of C16-α-bromoamide probe (3)**

α-Bromopalmitamide **3** was synthesized using a modified procedure of our prior work [29]. In short, 2-azidoethanamine was coupled to PMB-protected pantothenate, followed by reduction of the terminal azide to the amine. PyBOP-catalyzed peptide bond formation between commercially available 2-bromopalmitate and the free amine followed by deprotection afforded compound **3**.

**Synthesis of 2-azidoethanamine (S2)**


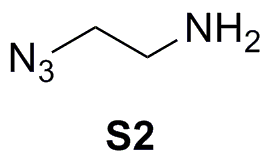


2-Bromoethylamine hydrobromide (2.0 g, 9.76 mmol) was added to a solution of sodium azide (1.9 g, 29.3 mmol) in H_2_O (10 mL). The stirred solution was heated to 75 °C for 24 h before it was cooled to 0 °C. Et_2_O (10 mL) was added followed by solid KOH (3 g). The organic phase was separated and the aqueous layer extracted with Et_2_O (3x50 mL). The combined organic layers were dried with Na_2_SO_4_ and the solvent carefully removed by air flow. The colorless oil was dried overnight in a desiccator (90%) to afford azide **S2**. ^1^H NMR (400 MHz, CDCl_3_): δ = 1.68 (s), 2.87 (t), 3.36 (t) ppm. ^13^C NMR (100 MHz, CDCl_3_): δ = 41.45, 54.73 ppm. ESI-MS [M+H]^+^ calcd. 86.06, found 86.10.

**Synthesis of 3-((4*R*)-2-(4-methoxyphenyl)-5,5-dimethyl-1,3-dioxane-4-carboxamido)propanoic acid (S3)**


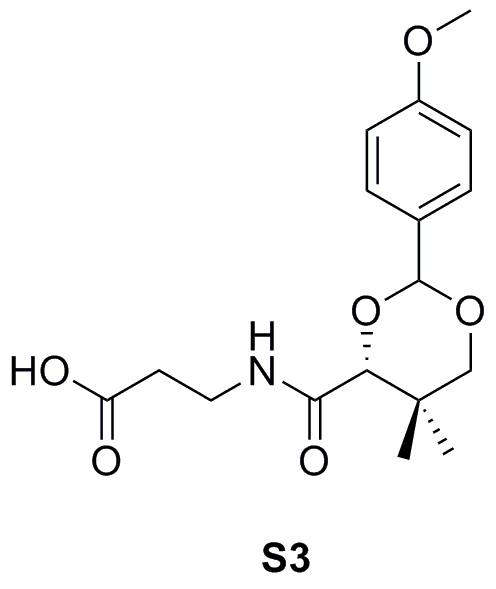


D-Pantothenic acid hemicalcium salt (10 g, 42.0 mmol) was dissolved in anhydrous DMF (50 mL). Concentrated H_2_SO_4_ (1.3 mL, 42 mmol) was added dropwise and stirred for 30 minutes. p-Anisaldehyde dimethyl acetal (7.2 mL, 42 mmol) and CSA (1.0 g, 4.6 mmol) were added and the reaction was stirred for 16 h. The reaction mixture was partitioned between EtOAc (250 mL) and H_2_O (250 mL). The organic layer was washed with saturated NaHCO_3_ (5x) and brine (5x). The organic layer was dried (Na_2_SO_4_) and evaporated. The resulting white solid was washed with cold dichloromethane to remove any remaining p-anisaldehyde dimethyl acetal to yield the desired product as a white crystalline solid (75%). ^1^H-NMR (CDCl_3_, 400 MHz): δ= 9.87 (s), 7.41 (d), 6.91 (d), 5.45 (s), 4.10 (s), 3.79 (s), 3.69-3.49 (m), 2.59 (t), 1.09 (s), 1.08 (s). ^13^C-NMR (CDCl_3_, 100 MHz): δ= 177.03, 169.75, 160.41, 130.40, 127.64, 113.96, 101.42, 83.90, 78.68, 55.52, 34.31, 34.03, 33.33, 22.00, 19.31. ESI-MS [M+H]^+^ calcd. 337.15, found 337.20.

**Synthesis of (4*R*)-N-(3-(2-azidoethylamino)-3-oxopropyl)-2-(4-methoxyphenyl)-5,5-dimethyl-1,3-dioxane-4-carboxamide (S4)**

**
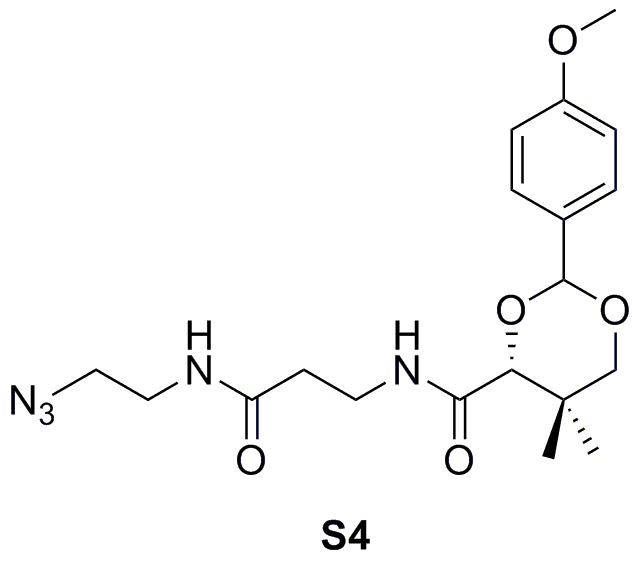
**

Pantothenate **S3** (3.3 g, 9.76 mmol), 2-azidoethanamine (840 mg, 9.76 mmol), EDC (3.7 g, 19.5 mmol), and HOBt (3.0 g, 19.5 mmol) were combined and dissolved in dry DMF (10 mL). DIPEA (3.4 mL, 19.5 mmol) was added, and the reaction was allowed to stir overnight. The reaction mixture was partitioned between EtOAc and H_2_O and the organic phase washed with saturated NaHCO_3_ (3 x 50 mL) and brine (3 x 50 mL). Elution through a plug of Si-gel with EtOAc gave the pure product **S4** as a yellow oil (75%). ^1^H-NMR (400 MHz, CDCl_3_) δ 7.36 (d), 7.06 (bt), 6.93 (bt), 6.85 (d), 5.39 (s), 3.99 (s), 3.73 (s), 3.62 (q), 3.46 (m), 3.26 (m), 2.37 (t), 1.04 (s), 1.01 (s). ^13^C-NMR (100 MHz, CDCl_3_) δ 171.62, 169.74, 160.40, 130.34, 127.74, 113.89, 101.49, 84.00, 78.57, 55.50, 39.06, 35.88, 35.08, 33.22, 22.02, 19.31. ESI-MS [M+H]^+^ calcd. 406.20, found 406.20.

**Synthesis of (4*R*)-N-(3-(2-aminoethylamino)-3-oxopropyl)-2-(4-methoxyphenyl)-5,5-dimethyl-1,3-dioxane-4-carboxamide (S5)**


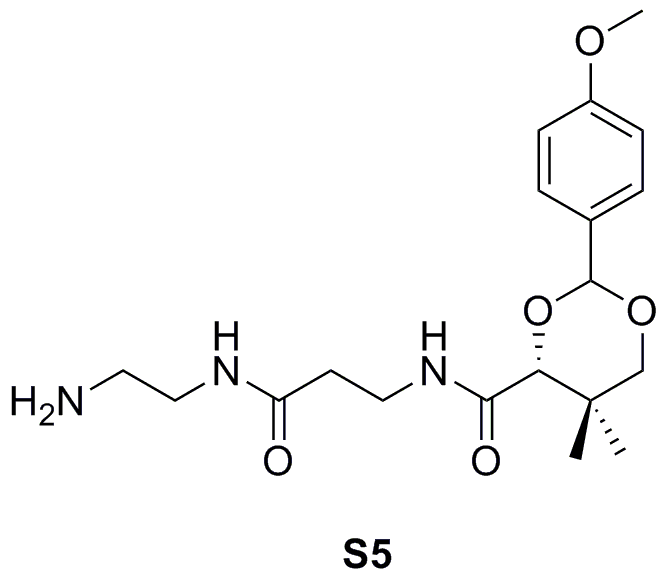


To a solution of azide **S4** (2.9 g, 7.2 mmol) in THF (10 mL), PPh_3_ (2.1 g, 8.0 mmol), and water (1 ml) were added and stirred at room temperature for 24 h. Evaporation of the solvent under reduced pressure gave oil from which amine **S5** was isolated by flash chromatography (CH_2_Cl_2_ to 15% MeOH/CH_2_Cl_2_/1.5% Et_3_N) as a golden oil (70%). ^1^H-NMR (400 MHz, CDCl_3_) δ 7.41 (d), 7.09 (bt), 6.92 (d), 6.54 (bt), 5.45 (s), 4.07 (s), 3.81 (s), 3.68 (q), 3.55 (m), 3.25 (m), 2.77 (t), 2.63 (q), 2.43 (t), 1.89 (bs), 1.10 (s), 1.08 (s). ^13^C-NMR (100 MHz, CDCl_3_) δ 172.53, 170.10, 160.34, 130.28, 127.74, 113.87, 101.44, 83.96, 78.48, 55.46, 40.63, 35.80, 35.50, 33.15, 21.89, 19.22. ESI-MS [M+H]^+^ calcd. 380.21, found 380.30.

**Synthesis of (4*R)*-N-(3-(2-(2-bromohexadecanamido)ethylamino)-3-oxopropyl)-2-(4-methoxyphenyl)-5,5-dimethyl-1,3-dioxane-4-carboxamide (S6)**


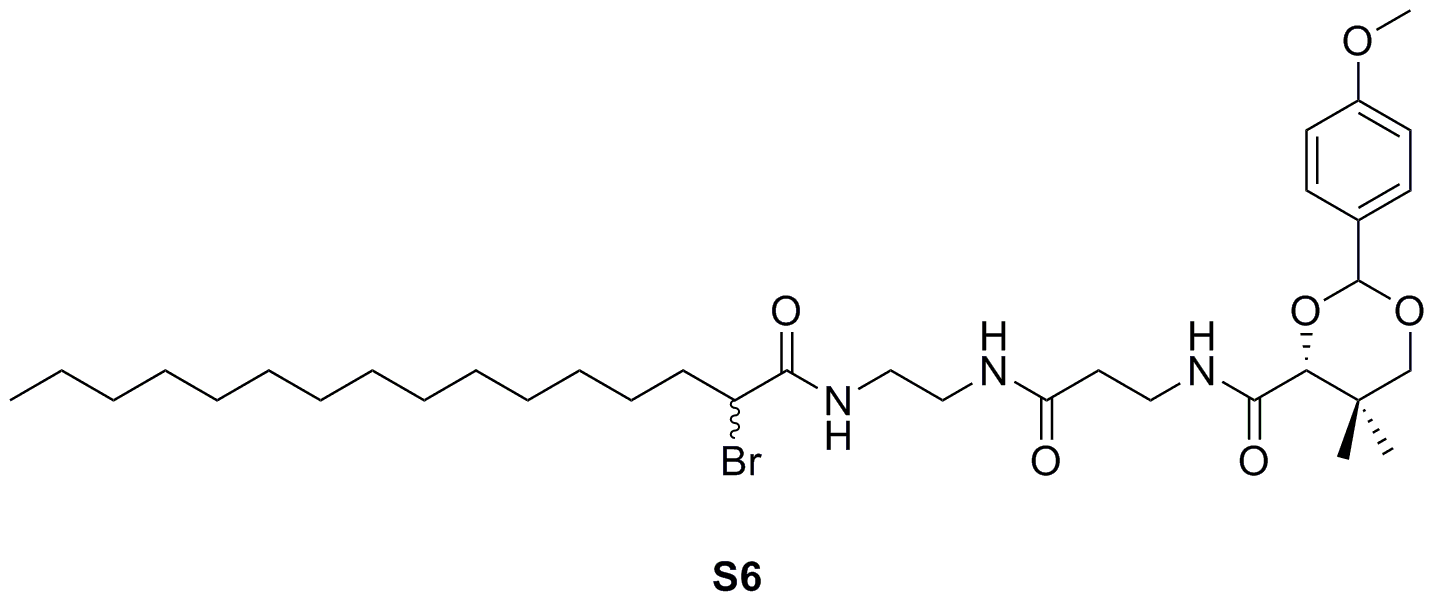


Amine **S5** (500 mg, 1.32 mmol), PyBOP (754 mg, 1.45 mmol), and α-bromopalmitate (442 mg, 1.32 mmol) were combined and dissolved in dry CH_2_Cl_2_ (25 mL). DIPEA (460 μL, 2.64 mmol) was added at 0 °C and stirred overnight. The solvent was removed under reduced pressure, and the resultant oil was purified by column chromatography (CH_2_Cl_2_ to 5% CH_2_Cl_2_/MeOH) to yield compound **S6** as a white solid (90%). ^1^H-NMR (400 MHz, CDCl_3_) δ 7.41 (d), 7.09 (bs), 6.89 (d), 5.43 (s), 4.19 (m), 4.04 (s), 3.77 (s), 3.64 (q), 3.50 (m), 3.30 (m), 2.88 (bs), 2.39 (t), 2.01 (bs), 1.22 (bs), 1.06 (s), 1.03 (s), 0.85 (t). ^13^C-NMR (100 MHz, CDCl_3_) δ 172.53, 170.65, 170.06, 160.44, 130.27, 127.80, 113.94, 101.55, 83.98, 78.57, 55.54, 50.69, 40.16, 39.31, 36.32, 35.67, 35.31, 33.27, 32.15, 29.94-29.60, 29.17, 27.62, 22.93, 22.08, 19.36, 14.38. ESI-MS [M+H]^+^ calcd. 696.35, found 696.50.

**Synthesis of (*R*)-2-bromo-N-(2-(3-(2,4-dihydroxy-3,3-dimethylbutanamido)propanamido)ethyl)hexadecanamide (3)**


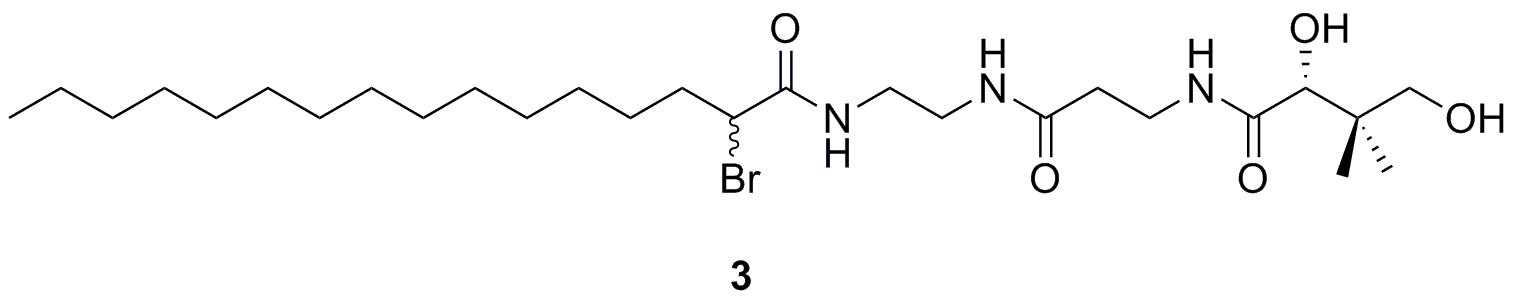


PMB-protected bromopalmitoyl pantetheine **S6** (50 mg, 0.07 mmol) was dissolved in 10 mL dioxane and cooled. An equivalent volume of 1 M HCl (aq) was added dropwise. The reaction was allowed to slowly attain room temperature and stirred for 2 h. Once the reaction was complete, solid NaHCO_3_ was added to the reaction mixture until the solution was neutral. The mixture was evaporated and extracted with CH_2_Cl_2_, the solvent evaporated and the crude product purified by column chromatography (CH_2_Cl_2_ to 20% MeOH/CH_2_Cl_2_) to yield the white solid product as a mixture of diastereomers (80%). ^1^H-NMR (400 MHz, (CD_3_)_2_SO) δ 8.27 (bt), 7.90 (bt), 7.69 (bt), 5.35 (d), 4.45 (t), 4.27 (t), 3.68 (d), 3.29-3.05 (m), 2.48 (bs), 2.23 (t), 1.21 (bs), 0.85 (t), 0.78 (s), 0.75 (s). ^13^C-NMR (100 MHz, (CD_3_)_2_SO) δ 173.51, 171.37, 169.23, 86.94, 75.70, 68.72, 50.32, 39.24, 38.62, 35.88, 35.46, 35.28, 31.98, 29.73-29.40, 28.94, 27.46, 22.78, 21.59, 20.98, 14.63. ESI-MS [M+H]^+^ calcd. 578.31, found 578.40.

**Summary of synthesis of C6-α-bromoamide probe (6)**

The synthesis of α-bromopalmitamide **6** was achieved by PyBOP-catalyzed peptide bond formation between the free amine **S5** and commercially available 2-bromohexanoic acid to afford **S7**, followed by deprotection.

**Synthesis of (4*R*)-N-(3-(2-(2-bromohexanamido)ethylamino)-3-oxopropyl)-2-(4-methoxyphenyl)-5,5-dimethyl-1,3-dioxane-4-carboxamide (S7)**

**
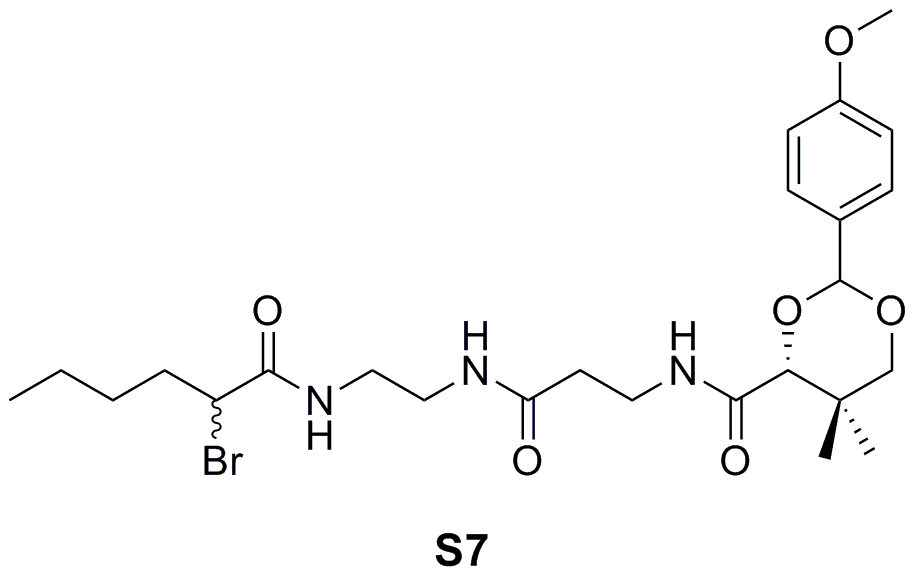
**

Amine **S5** (250 mg, 0.66 mmol), PyBOP (343 mg, 0.66 mmol), and 2-bromohexanoic acid (94 μl, 0.66 mmol) were combined and dissolved in dry CH_2_Cl_2_ (15 mL). DIPEA (230 μL, 1.32 mmol) was added at 0 °C and stirred overnight. The reaction mixture was extracted with saturated aqueous citric acid, the solvent removed under reduced pressure, and the resultant oil was purified by column chromatography (CH_2_Cl_2_ to 10% MeOH/CH_2_Cl_2_) to yield compound **S7** as a golden clear oil (90%). ^1^H-NMR (400 MHz, CDCl_3_) δ 7.42 (d), 7.22 (bs), 7.01 (bt), 6.90 (d), 6.81 (bs), 5.44 (s), 4.20 (m), 4.06 (s), 3.80 (s), 3.67 (q), 3.52 (bq), 3.34 (bs), 3.26 (bm), 2.41 (t), 2.04 (bm), 1.96 (bm), 1.32 (bm), 1.09 (s), 1.06 (s), 0.88 (t). ^13^C-NMR (100 MHz, CDCl_3_) δ 172.20, 170.26, 169.87, 160.47, 130.36, 127.76, 113.96, 101.56, 84.07, 78.66, 55.56, 50.99, 40.56, 39.50, 36.40, 35.52, 35.22, 33.30, 29.62, 22.19, 22.10, 19.38, 14.07. ESI-MS [M+H]^+^ calcd. 556.19, found 556.30.

**Synthesis of (*R*)-2-bromo-N-(2-(3-(2,4-dihydroxy-3,3-dimethylbutanamido)propanamido)ethyl)hexanamide (6)**


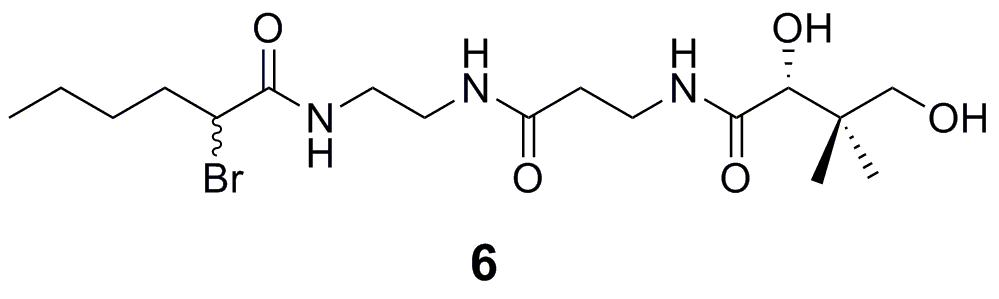


PMB-protected bromohexanoyl pantetheine **S7** (50 mg, 0.09 mmol) was dissolved in 10 mL dioxane and cooled. An equivalent volume of 1 M HCl (aq) was added dropwise. The reaction was allowed to slowly attain room temperature and stirred for 4 h. Once the reaction was complete, solid NaHCO_3_ was added to the reaction mixture until the solution was neutral. The mixture was evaporated and the crude product purified by column chromatography (CH_2_Cl_2_ to 20% MeOH/CH_2_Cl_2_) to yield the product as a mixture of diastereomers (80%). ^1^H-NMR (400 MHz, (CD_3_)_2_SO) δ 8.28 (bt), 7.89 (bt), 7.69 (bt), 5.35 (d), 4.45 (t), 4.27 (t), 3.68 (d), 3.29-3.05 (m), 2.48 (bs), 2.23 (t), 1.90 (m), 1.83 (m), 1.27 (m), 0.85 (t), 0.78 (s), 0.75 (s). ^13^C-NMR (100 MHz, (CD_3_)_2_SO) δ 173.51, 171.39, 169.24, 75.72, 68.72, 50.30, 39.24, 38.62, 35.90, 35.47, 35.03, 29.65, 22.14, 21.60, 20.99, 14.43. ESI-MS [M+H]^+^ calcd. 438.15, found 438.20.

**
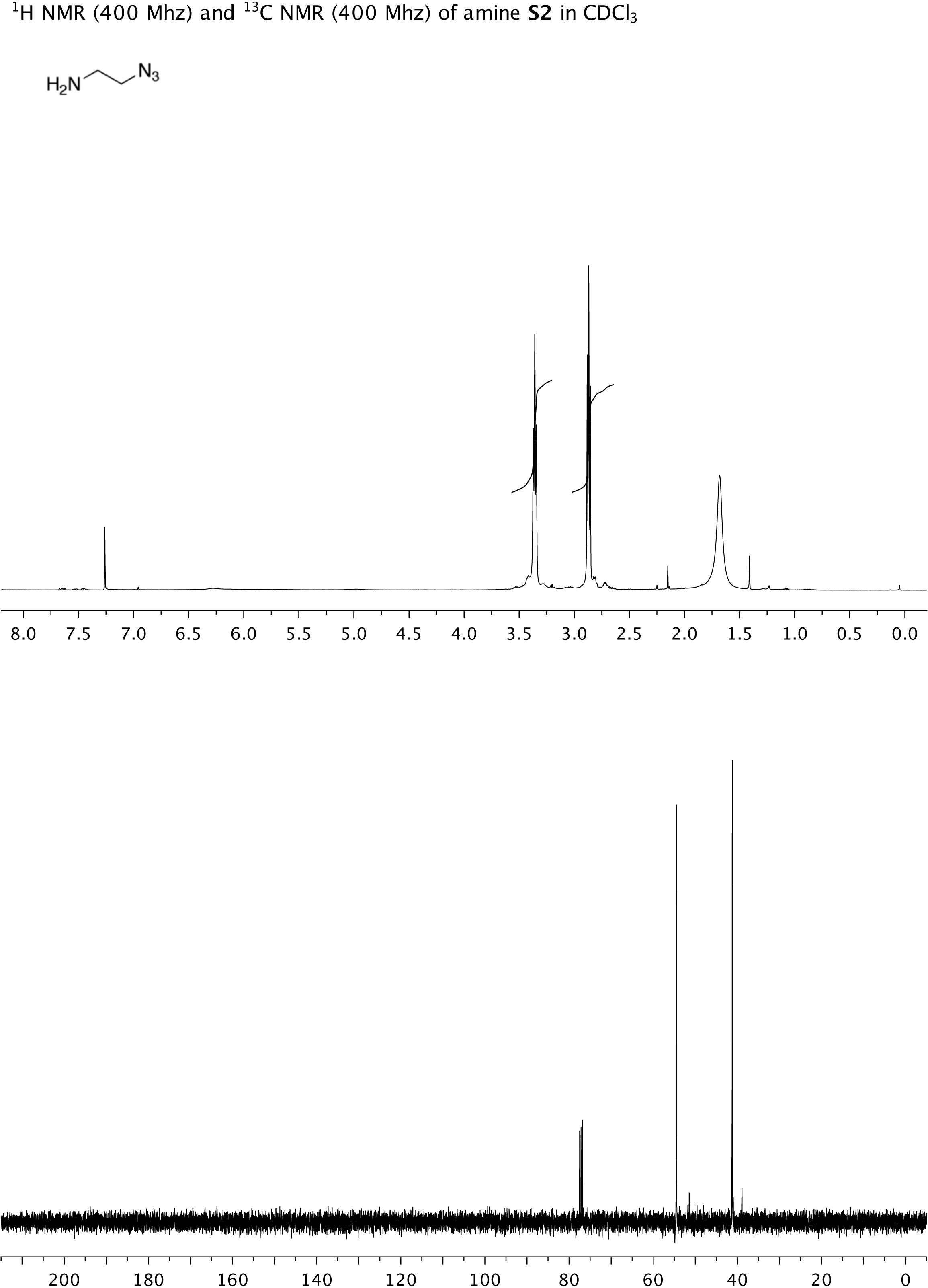
**

**
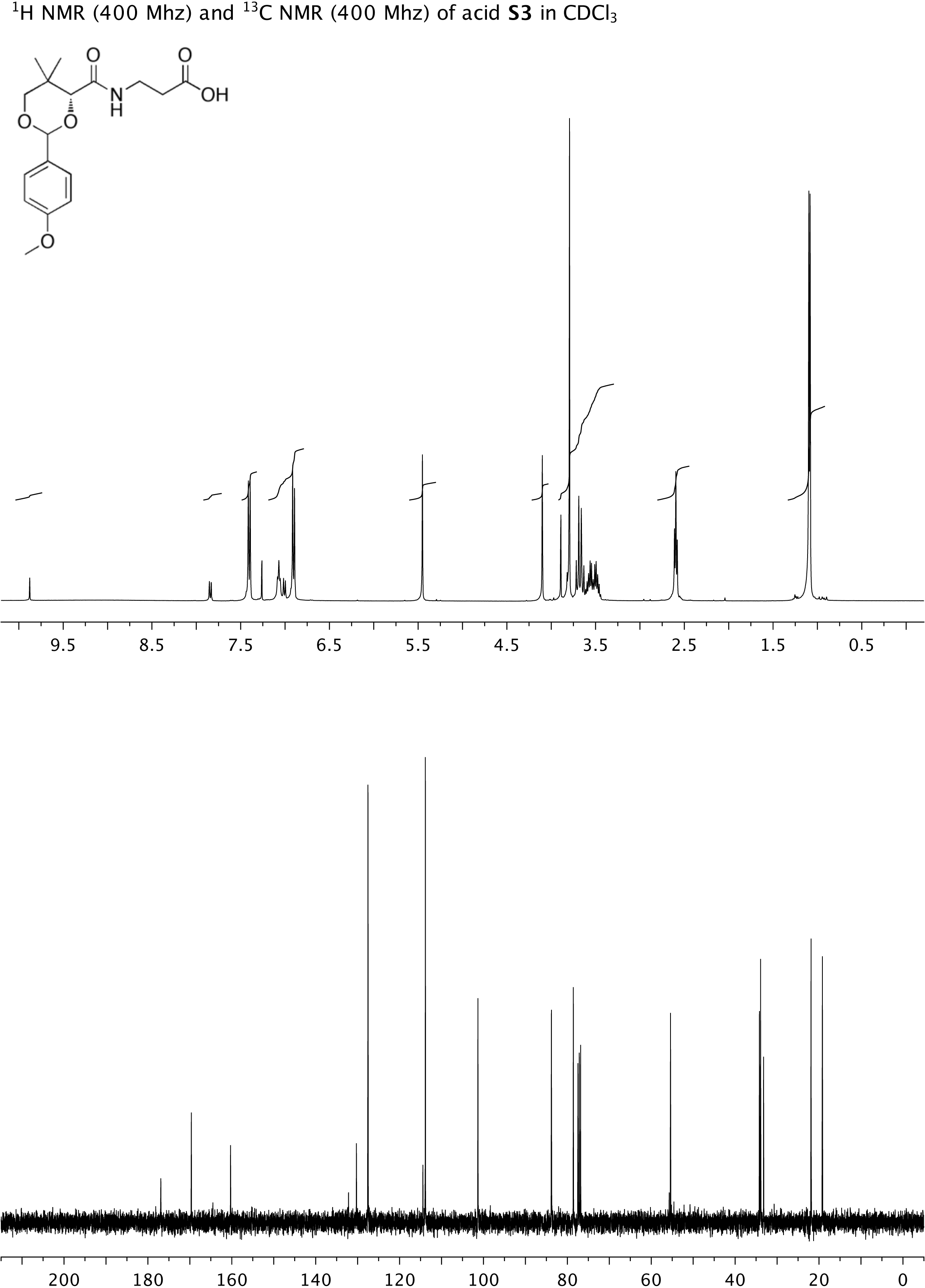
**

**
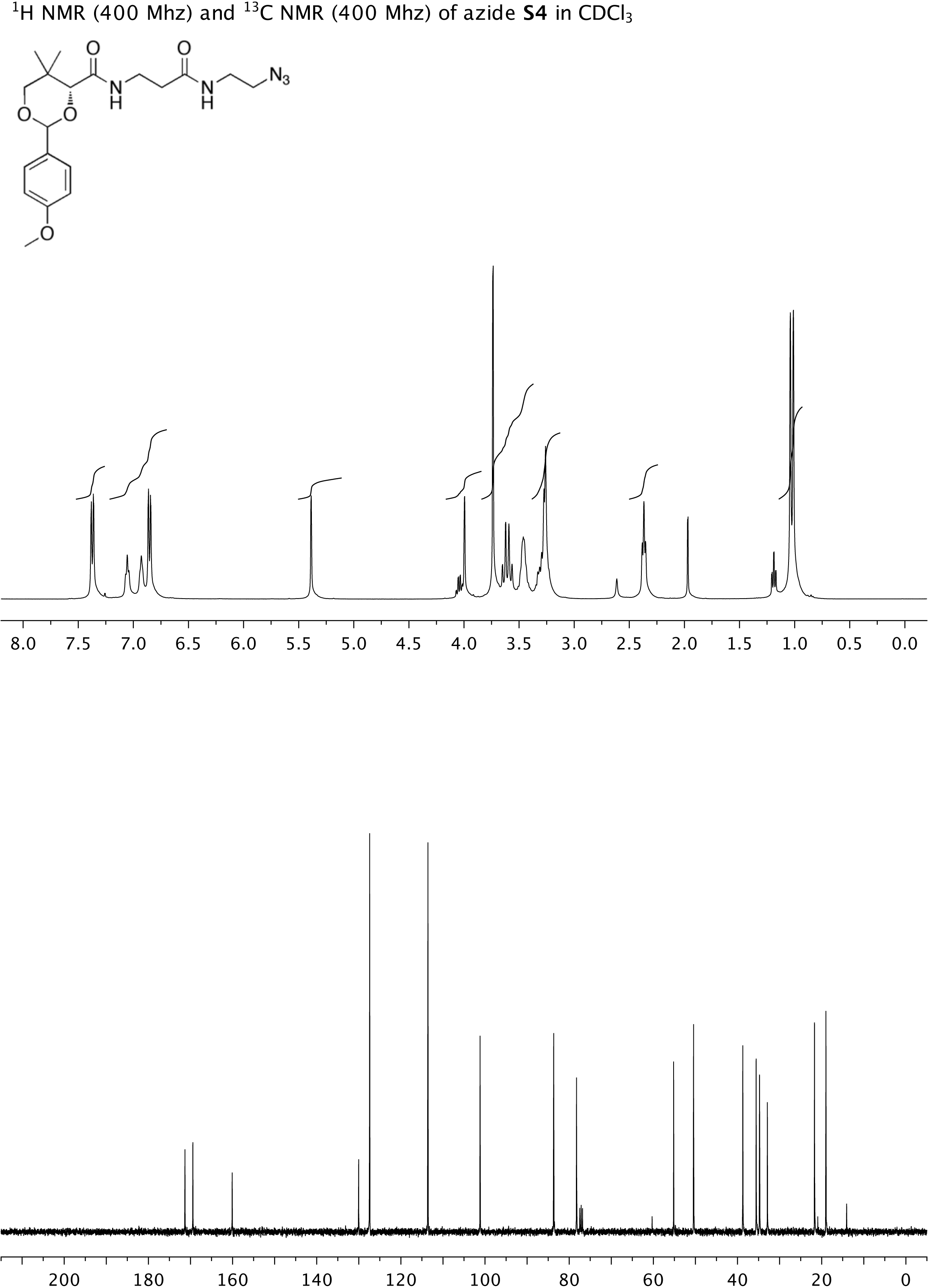
**

**
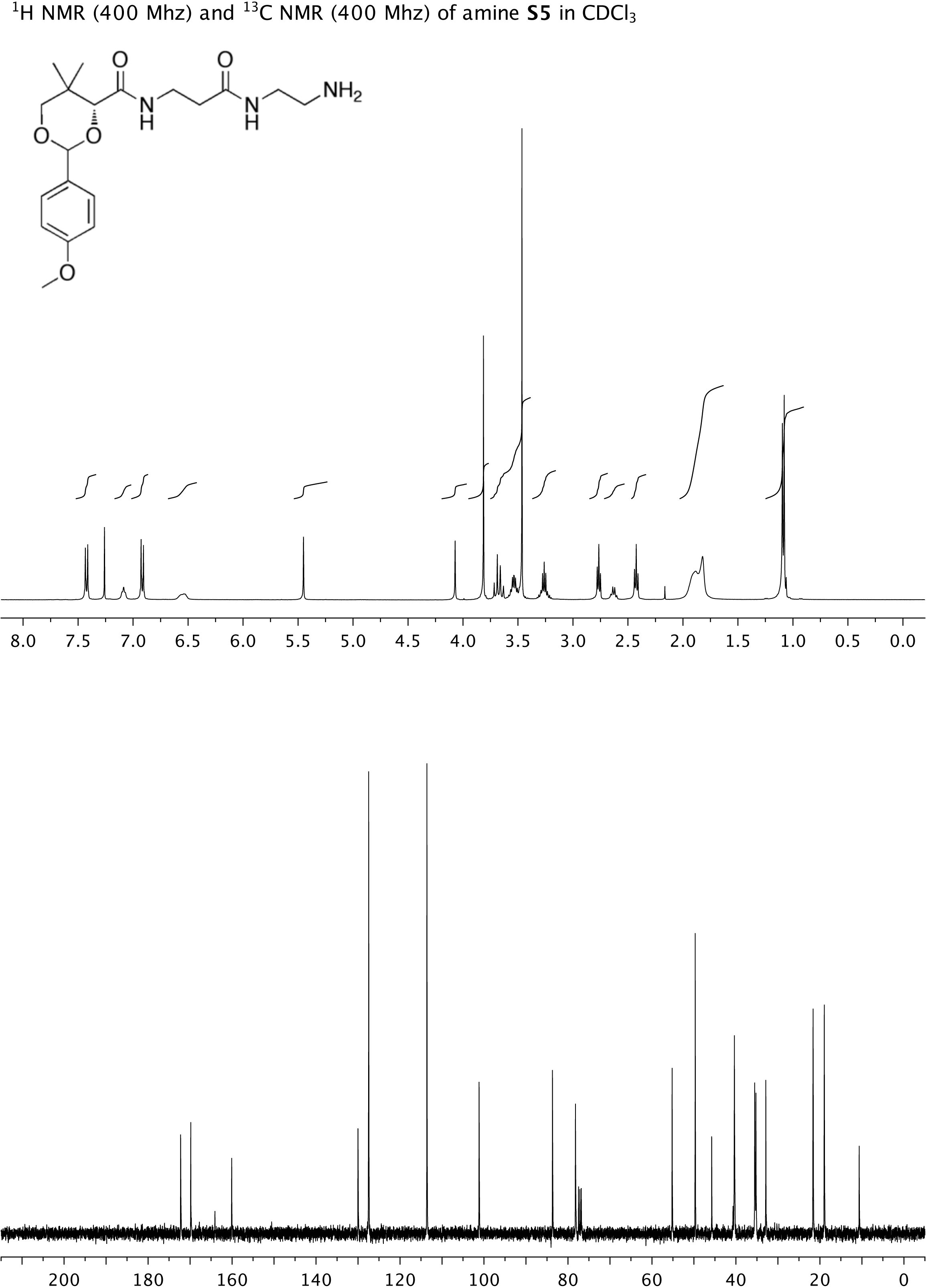
**

**
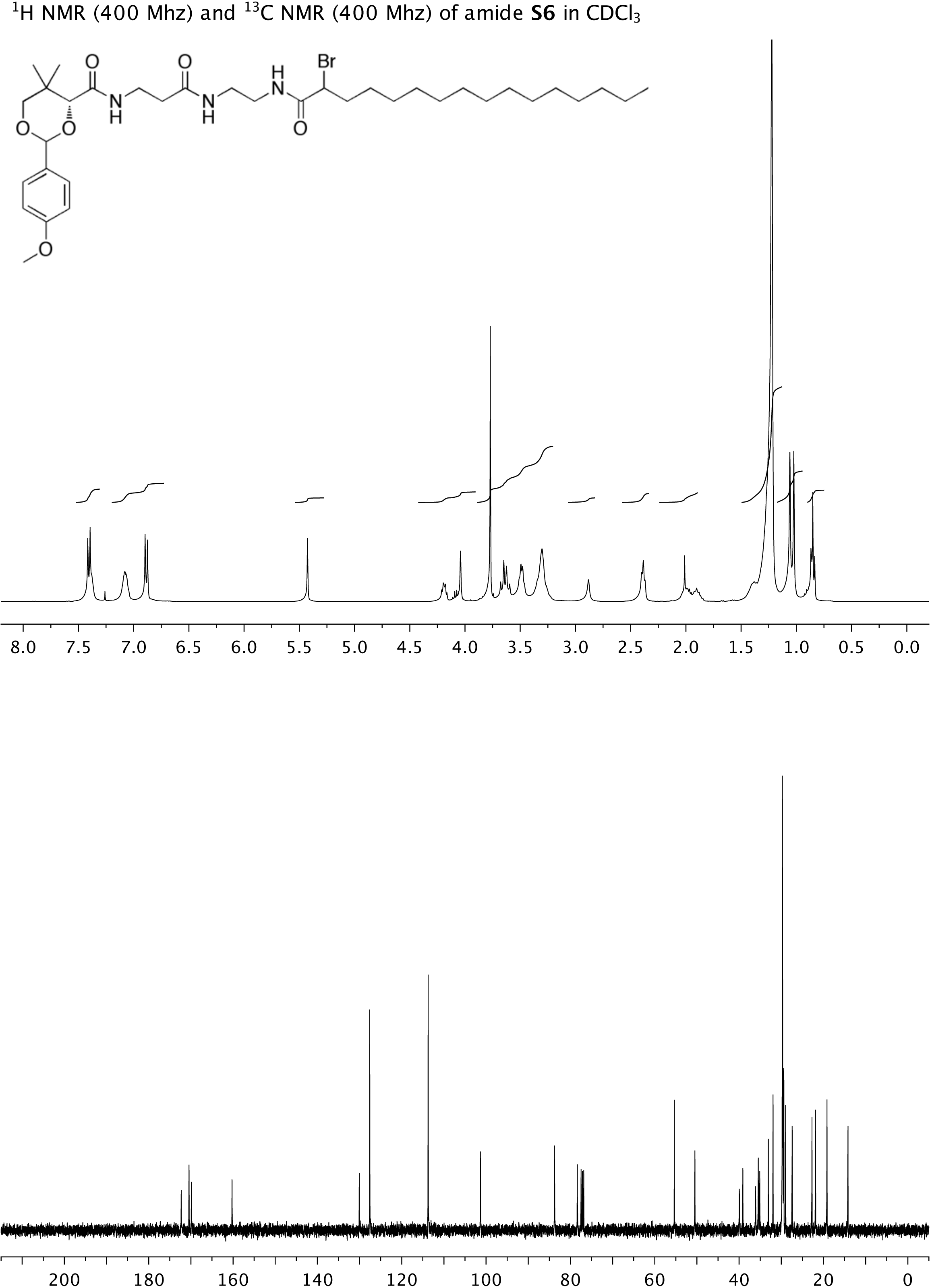
**

**
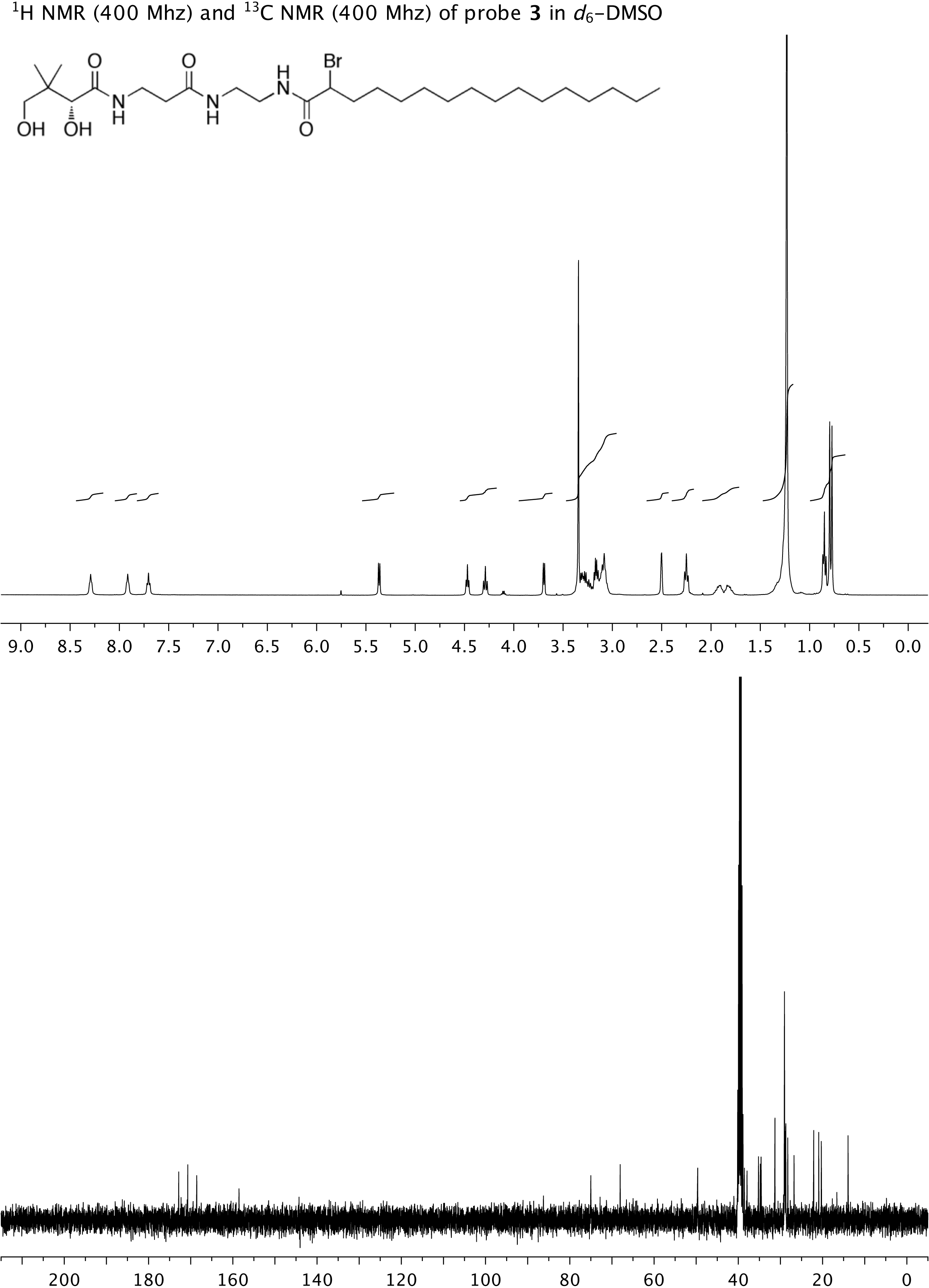
**

**
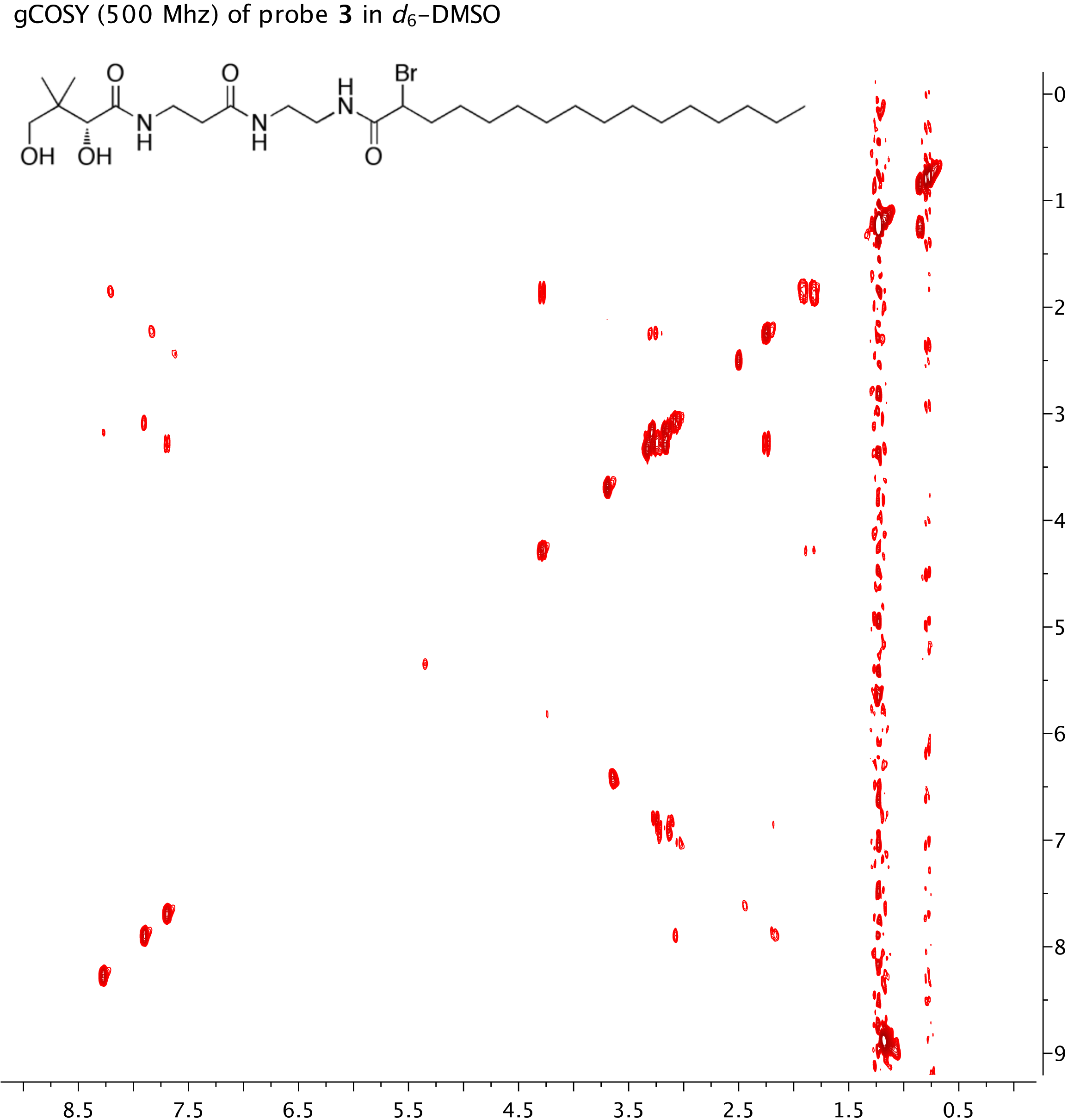
**

**
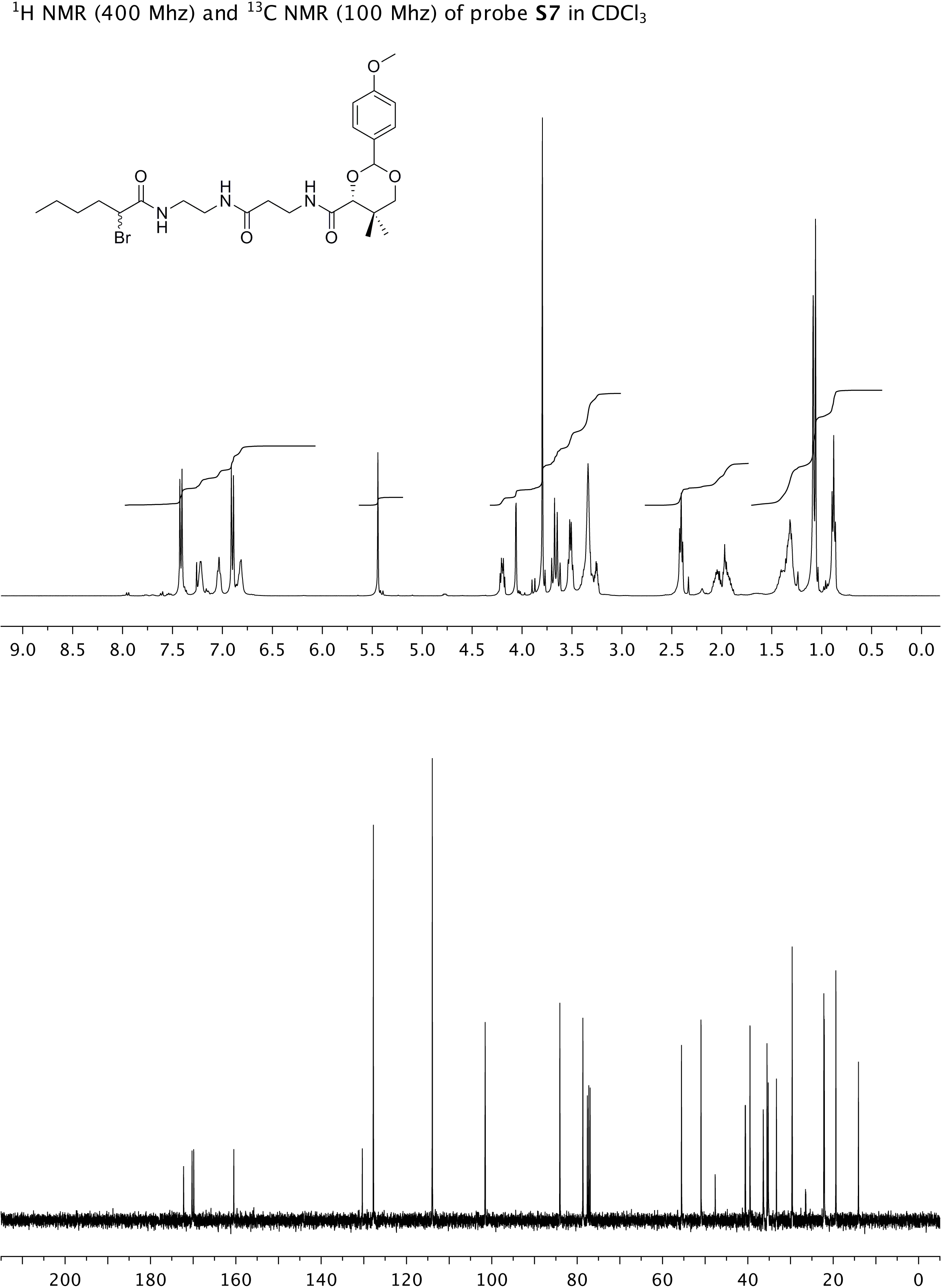
**

**
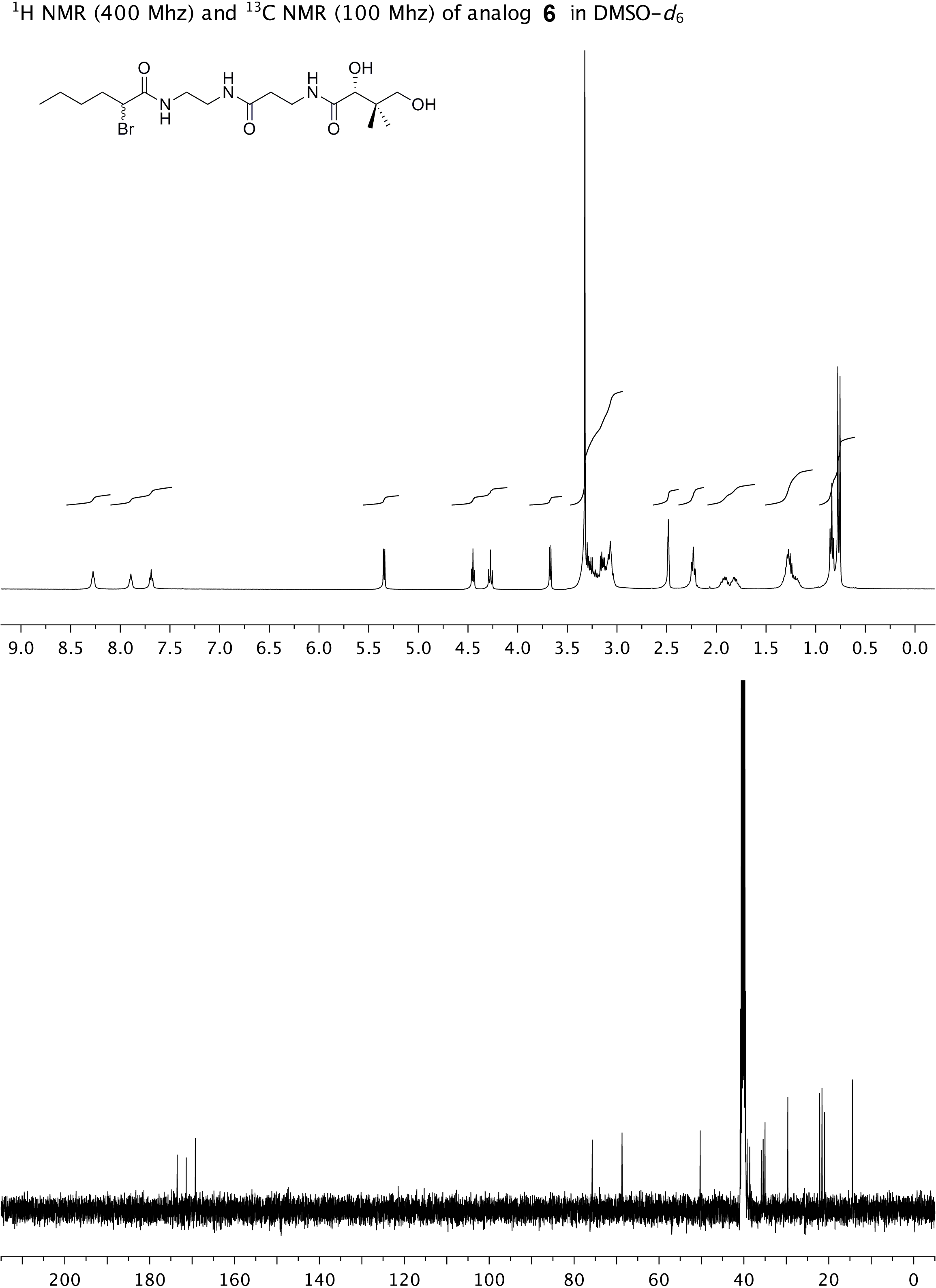
**

**References**

1. Armougom F, Moretti S, Poirot O, Audic S, Dumas P, et al. (2006) Expresso: automatic incorporation of structural information in multiple sequence alignments using 3D-coffee. Nucleic Acids Research 34: W604-W608.

2. Gouet P, Courcelle E, Stuart DI, Metoz F (1999) ESPript: analysis of multiple sequence alignments in PostScript. Bioinformatics 15: 305-308.

3. Worthington AS, Burkart MD (2006) One-pot chemo-enzymatic synthesis of reporter-modified proteins. Organic & Biomolecular Chemistry 4: 44-46.

4. Dereeper A, Guignon V, Blanc G, Audic S, Buffet S, et al. (2008) Phylogeny.fr: robust phylogenetic analysis for the non-specialist. Nucleic Acids Research 36: W465-W469.

5. Arnold K, Bordoli L, Kopp J, Schwede T (2006) The SWISS-MODEL workspace: a web-based environment for protein structure homology modelling. Bioinformatics 22: 195-201.

6. Roy A, Kucukural A, Zhang Y (2010) I-TASSER: a unified platform for automated protein structure and function prediction. Nature Protocols 5: 725-738.

7. Comeau SR, Gatchell DW, Vajda S, Camacho CJ (2004) ClusPro: An automated docking and discrimination method for the prediction of protein complexes. Bioinformatics 20: 45-50.

8. PyMol (v1.4) Schrödinger, LLC.

9. Morris GM, Goodsell DS, Huey R, Olson AJ (1996) Distributed automated docking of flexible ligands to proteins: Parallel applications of AutoDock 2.4. Journal of Computer-Aided Molecular Design 10: 293-304.

10. Gordon JC, Myers JB, Folta T, Shoja V, Heath LS, et al. (2005) H++: a server for estimating pK(a)s and adding missing hydrogens to macromolecules. Nucleic Acids Research 33: W368-W371.

11. Schuttelkopf AW, van Aalten DMF (2004) PRODRG: a tool for high-throughput crystallography of protein-ligand complexes. Acta Crystallographica Section D 60: 1355-1363.

12. Sanner MF (1999) Python: A programming language for software integration and development. Journal of Molecular Graphics & Modelling 17: 57-61.

13. Nielsen H, Engelbrecht J, Brunak S, vonHeijne G (1997) Identification of prokaryotic and eukaryotic signal peptides and prediction of their cleavage sites. Protein Engineering 10: 1-6.

14. Emanuelsson O, Nielsen H, Von Heijne G (1999) ChloroP, a neural network-based method for predicting chloroplast transit peptides and their cleavage sites. Protein Science 8: 978-984.

15. Claros MG, Vincens P (1996) Computational method to predict mitochondrially imported proteins and their targeting sequences. European Journal of Biochemistry 241: 779-786.

16. Small I, Peeters N, Legeai F, Lurin C (2004) Predotar: A tool for rapidly screening proteomes for N-terminal targeting sequences. Proteomics 4: 1581-1590.

17. Peeters N, Small I (2001) Dual targeting to mitochondria and chloroplasts. Biochimica et Biophysica Acta 1541: 54-63.

18. Harris EH (1989) The Chlamydomonas Sourcebook: A comprehensive guide to biology and laboratory use. San Diego, CA: Academic Press.

19. Mendez M, O'Neill B, Burkart MD, Behnke C, Lieberman S, et al. (2010) Production of fatty acids by genetically modified photosynthetic organisms. Patent IPN: WO2010/019813A2

20. Murugan E, Kong R, Sun H, Rao F, Liang Z-X (2010) Expression, purification and characterization of the acyl carrier protein phosphodiesterase from *Pseudomonas aeruginosa*. Protein Expression and Purification 71: 132-138.

21. Dormann P, Frentzen M, Ohlrogge JB (1994) Specificities of the acyl-acyl-carrier protein (ACP) thioesterase and glycerol-3-phosphate acyl transferase for octadecenoyl-ACP isomers - identification of a petroselinoyl-ACP thioesterase in Umbelliferae. Plant Physiology 104: 839-844.

22. Araki S, Sakurai T, Kawaguchi A, Murata N (1987) Positional distribution of fatty acids in glycerolipids of the marine red alga *Porphyra yezoensis*. Plant and Cell Physiology 28: 761-766.

23. Jones A, Davies HM, Voelker TA (1995) Palmitoyl-acyl carrier protein (ACP) thioesterase and the evolutionary origin of plant acyl-ACP thioesterases. Plant Cell 7: 359-371.

24. Mayfield SP, Franklin SE, Lerner RA (2003) Expression and assembly of a fully active antibody in algae. Proceedings of the National Academy of Sciences, USA 100: 438-442.

25. Boynton JE, Gillham NW, Harris EH, Hosler JP, Johnson AM, et al. (1988) Chloroplast transformation in Chlamydomonas with high velocity microprojectiles. Science 240: 1534-1538.

26. Gorman DS, Levine RP (1965) Cytochrome F and plastocyanin - their sequence in photosynthetic electron transport chain of *Chlamydomonas reinhardtii*. Proceedings of the National Academy of Sciences, USA 54: 1665-1669.

27. Kan Y, Pan J (2010) A one-shot solution to bacterial and fungal contamination in the green alga *Chlamydomonas reinhardtii* culture by using an antibiotic cocktail. Journal of Phycology 46: 1356-1358.

28. Worthington AS, Hur GH, Meier JL, Cheng Q, Moore BS, et al. (2008) Probing the compatibility of type II ketosynthase-carrier protein partners. ChemBioChem 9: 2096-2103.

29. Worthington AS, Rivera H, Jr., Torpey JW, Alexander MD, Burkart MD (2006) Mechanism-based protein crosslinking probes to investigate carrier protein-mediated biosynthesis. ACS Chemical Biology 1: 687-691.
